# Sacrolide A, a new antimicrobial and cytotoxic oxylipin macrolide from the edible cyanobacterium *Aphanothece sacrum*

**DOI:** 10.3762/bjoc.10.190

**Published:** 2014-08-07

**Authors:** Naoya Oku, Miyako Matsumoto, Kohsuke Yonejima, Keijiroh Tansei, Yasuhiro Igarashi

**Affiliations:** 1 Biotechnology Research Center and Department of Biotechnology, Toyama Prefectural University, 5180 Kurokawa, Imizu, Toyama 939-0398, Japan; 2 Suizenjinori-Hompo Tanseidoh, 5-13-3 Tsuboi, Chuo-ku, Kumamoto, Kumamoto 860-0863, Japan

**Keywords:** *Aphanothece sacrum*, cyanobacterium, food intoxication, natural products, sacrolide A, *suizenji-nori*

## Abstract

Macroscopic gelatinous colonies of freshwater cyanobacterium *Aphanothece sacrum*, a luxury ingredient for Japanese cuisine, were found to contain a new oxylipin-derived macrolide, sacrolide A (**1**), as an antimicrobial component. The configuration of two chiral centers in **1** was determined by a combination of chiral anisotropy analysis and conformational analysis of different ring-opened derivatives. Compound **1** inhibited the growth of some species of Gram-positive bacteria, yeast *Saccharomyces cerevisiae* and fungus *Penicillium chrysogenum*, and was also cytotoxic to 3Y1 rat fibroblasts. Concern about potential food intoxication caused by accidental massive ingestion of *A. sacrum* was dispelled by the absence of **1** in commercial products. A manual procedure for degrading **1** in raw colonies was also developed, enabling a convenient on-site detoxification at restaurants or for personal consumption.

## Introduction

Cyanobacteria continue to be core sources for bioactive secondary metabolites [1–2], and their significance in drug discovery has increased for the past two decades [3]. Some cyanobacteria form macroscopic colonies and are harvested for human consumption in many parts of the world [4].

As a part of our program to exploit untapped microbes for drug discovery, we focus on cyanobacteria that are eaten in Japan, and previously reported the isolation of an unusual ω-1 fatty acid (9*Z*,12*Z*)-9,12,15-hexadecatrienoic acid from a freshwater periphytic cyanobacterium *Nostoc verrucosum* [4].

*Aphanothece sacrum* is also an edible cyanobacterium, which is an endemic species in the Aso water system in the Kyushu District, Japan. It inhabits oligotrophic but mineral-rich freshwater such as spring-fed ponds with a narrow temperature range of 18 °C–20 °C [5] and forms thin crispate gelatinous floating colonies. The first record of this alga being consumed as food appeared 250 years ago. Various laver sheets, such as *suizenji-nori*, *akizuki-nori*, *jusentai*, or *shikintai*, were developed and designated as regional specialty goods by the local lords [6]. These products have also been presented as a tribute to the shogun family, and because of its economic value, the feudal domains protected habitats from overharvesting and water pollution. In 1924, the government designated the habitat in Kamiezuko Pond as a national treasure. However, with the increasing urbanization in this region, the supply of underground water has decreased significantly, which further narrowed the originally limited distribution of this species [7]. The only remaining natural habitat is reportedly in the Koganegawa Stream, a 2 km-long shallow stream where two companies engage in aquaculture [8]. *A. sacrum* is officially catalogued as a Class IA endangered species by the Ministry of Environment [9].

The aquaculture industry has supported chemical investigations of this rare alga. However, to date, only two substances have been reported: pseudovitamin B_12_ [10] and sulfated polysaccharide sacran [11].

**Figure 1:**
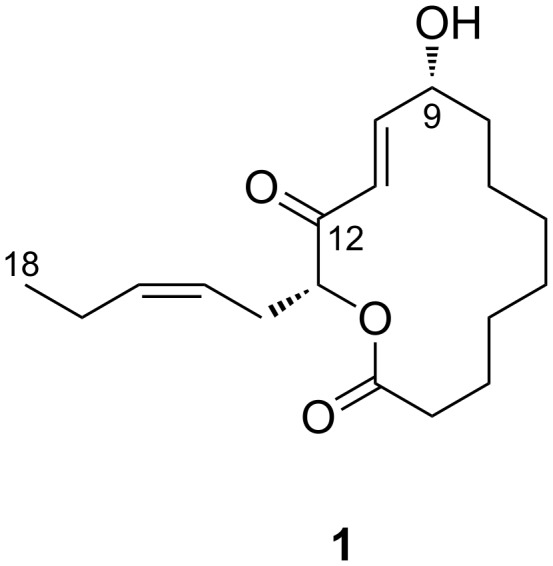
Structure of sacrolide A (**1**).

We evaluated the antimicrobial activity of this alga and found that a lipophilic fraction of an ethanolic extract showed inhibitory activity against several microbes. Activity-guided fractionation resulted in the discovery of a new macrolactonic oxylipin, sacrolide A (**1**, Figure 1).

## Results and Discussion

*A. sacrum* (500 g) was extracted with EtOH, and the combined extract was fractionated by solvent partitioning into *n*-hexane-, 10% aqueous MeOH-, 1-BuOH-, and water-soluble fractions. Antimicrobial testing against four Gram-positive bacteria (*Bacillus subtilis*, *Micrococcus luteus*, *Staphylococcus aureus*, and *Streptomyces lividans*), one Gram-negative bacterium (*Escherichia coli*), two yeasts (*Candida albicans* and *Saccharomyces cerevisiae*), and two fungi (*Aspergillus oryzae* and *Penicillum chrysogenum*) revealed that all these layers varied in the extent of activity against certain test organism(s). Especially intriguing was the antifungal activity exhibited by the *n*-hexane-soluble fraction, which, in most cases, comprises lipids. To identify the responsible constituent, the fraction was successively purified by silica gel chromatography, gel filtration on Sephadex LH-20, and finally by ODS HPLC to yield **1** (1.5 mg, 3.0 × 10^−4^% w/w). In addition, the antifungal principle in the aqueous MeOH fraction was isolated and identified as the same substance (6.0 mg, 1.2 × 10^−3^% w/w).

**Figure 2:**
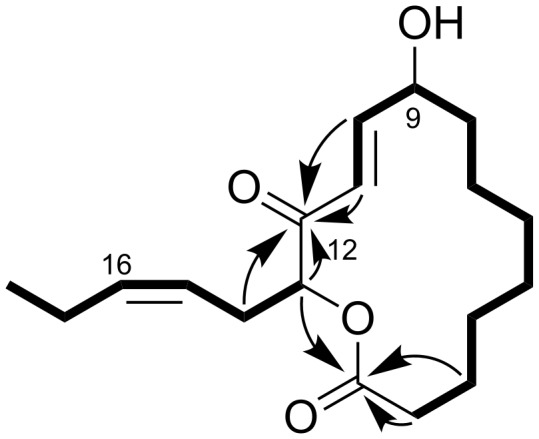
Selected COSY (bold lines) and HMBC (arrows) correlations for sacrolide A (**1**).

The molecular formula C_18_H_28_O_4_ was assigned to **1** based on a molecular ion *m/z* 331.1889 [M + Na]^+^ observed by HRMS (ESI) (calcd for C_18_H_28_NaO_4_, 331.1880). Of the five degrees of unsaturation calculated from the molecular formula, four were accounted for shielded carbonyl (δ_C_ 196.7, Table 1) and carboxyl (δ_C_ 172.9) groups, and two double bonds (δ_C_ 149.3, 135.6, 124.5, and 121.9) as observed in the ^13^C NMR spectrum. Thus, the remaining one degree of unsaturation is attributed to a cyclic structure. Because a resonance for a formyl proton (δ_H_ 9–10) was not observed in the ^1^H NMR spectrum, the carbonyl group must be part of a conjugated ketone. Other structural pieces were deduced from an HSQC spectrum to be two oxymethines (δ_H_/δ_C_ 5.23/77.3 and 4.50/71.1), nine methylenes (δ_H_/δ_C_ 1.74/33.81; 2.36, 2.50/33.82; 2.61, 2.56/28.7; 1.50/26.5; 1.35/26.4; 1.34/25.8; 1.68/24.3; 1.28/21.7; 2.06/20.8), and a methyl group (δ_H_/δ_C_ 0.97/14.1), which indicated that a lactone linkage is the only possible cyclization topology. The analysis of the COSY spectrum (Figure 2) helped trace six-carbon and ten-carbon spin systems, both of which include an oxymethine (H9: δ_H_ 4.50, H13: δ_H_ 5.23) and a disubstitued olefin. The geometry of the olefins was determined as *E* for Δ^10^ and *Z* for Δ^15^ based on the coupling constants *J*_H10,H11_ ~ 15.8 Hz and *J*_H15,H16_ ~ 10 Hz, respectively. The former spin system (O-H13-H_2_14-H15=H16-H_2_17-H_3_18) had a methyl group at one end and was therefore deemed to constitute a side chain group, whereas the latter (-H_2_2-H_2_3-H_2_4-H_2_5-H_2_6-H_2_7-H_2_8-H9(-O-)-H10=H11-) were bidirectionally opened by a methylene and a double bond at the termini and should constitute a body of the macrocycle core. The connectivity of the two fragments was verified by HMBC correlations from H10 (δ_H_ 6.93), H11 (δ_H_ 6.56), H13 (δ_H_ 5.23), and H_2_14 (δ_H_ 2.61, 2.56) to C12 (δ_C_ 196.7), forming an α,β-unsaturated α'-acyloxy ketone functionality, and from H_2_2 (δ_H_ 2.50, 2.36), H3 (δ_H_ 1.68), and H13 to C1 (δ_C_ 173.1), bridging a lactone linkage. The structure assembled to this point used up C_18_H_27_O_4_ of the molecular formula C_18_H_28_O_4_ and a remaining proton, not observed in the ^1^H spectrum, was assigned to a hydroxy proton (9-OH). Thus, the planar structure of **1** was determined to be a new 14-membered macrolide.

**Table 1:** ^1^H (500 MHz) and ^13^C (125 MHz) NMR data for sacrolide A (**1**) in CDCl_3_ (δ in ppm).

Position	δ_C_	δ_H_, mult. (*J* in Hz), integr.	HMBC (^1^H to ^13^C)

1	172.9		
2	33.81	2.36, m, 1H	1
		2.50, m, 1H	1
3	24.3	1.68, m, 2H	1, 2, 4, 5
4	25.8	1.34, m, 2H	2, 5
5	26.4	1.35, m, 2H	6
6	26.5	1.50, m, 2H	4, 5
7	21.7	1.28, m, 2H	4, 5, 6, 8, 9
8	33.82	1.74, m, 2H	5, 7, 9, 10
9	71.1	4.50, dt (4.7, 4.5), 1H	7, 8, 11
10	149.3	6.93, dd (4.7, 15.8), 1H	8, 9, 11, 12
11	124.5	6.56, d (15.8), 1H	9, 12
12	196.7		
13	77.3	5.23, dd (6.0, 7.6), 1H	1, 12, 14, 15
14	28.7	2.61, m, 2H	12, 13, 15, 16
		2.56, m, 1H	12, 13, 15, 16
15	121.9	5.30, td (7.9, 9.8), 1H	
16	135.6	5.54, td (7.6, 10.0), 1H	
17	20.6	2.06, qd (7.6, 7.4), 2H	15, 16, 18
18	14.1	0.97, t (7.6), 3H	16, 17

**Scheme 1:**
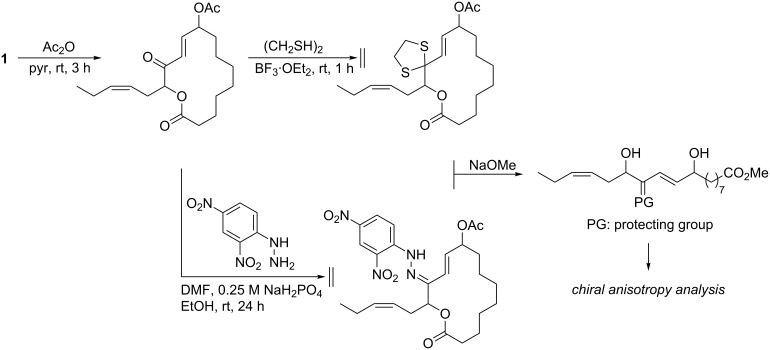
Initial derivatization strategy for the stereochemical analysis of sacrolide A (**1**).

**Scheme 2:**
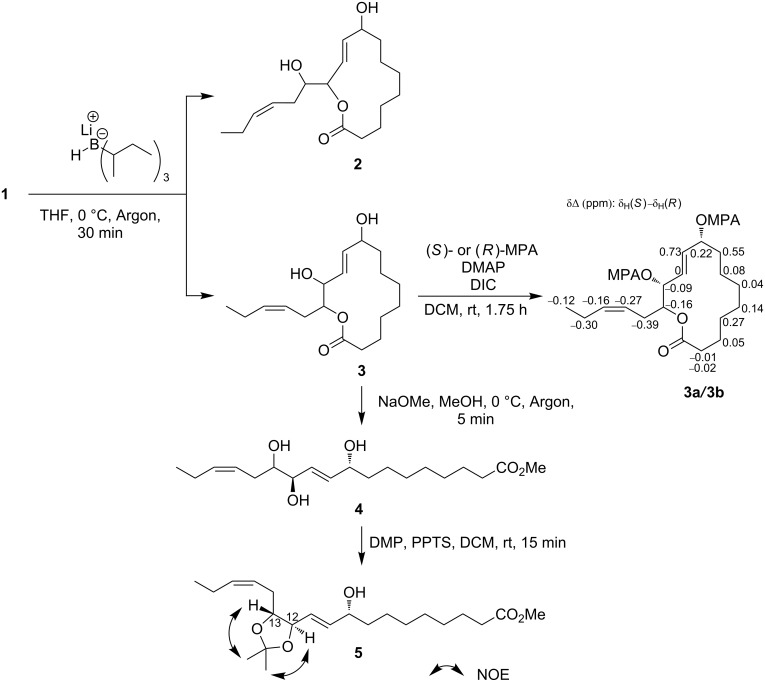
Determination of the stereochemistry of sacrolide A (**1**).

The stereogenic center C13 constitutes an α-acyloxy ketone moiety and deacylation catalyzed by a base may result in the loss of chirality because of epimerization [12]. To circumvent this problem, we initially attempted to protect the ketone group prior to chemical conversion (Scheme 1). Compound **1** was acetylated (Supporting Information File 1) and subjected to protection as 1,3-dithiane [13] or 1,4-dinitrophenylhydrazone [14]; however, neither of them resulted in the desired products. Then a strategy without protection was explored (Scheme 2). Reduction with L-selectride (lithium tri-*sec*-butylborohydride) gave the desired alcohol **3** and its ester-exchanged isomer **2** with a ratio of 2:1. To facilitate chiral anisotropy analysis at C9 and C12, the more abundant **3** was condensed either with (*S*)- or (*R*)-α-methoxyphenylacetic acid (MPA) [15] to give bis-MPA esters **3a** and **3b**. The δΔ values for the 2-en-1,4-diol moiety do not hold a diagnostic value because this region is under the influence of overlapping anisotropic effects by the flanking of two MPA residues. However, the consistent distribution of positive and negative values at the right half and on the side chain of the molecule was most reasonably explained by *R*-configurations for both the chiral centers. Thus, the (9*R*,12*R*)-configuration was established for **3**.

To correlate the *R*-configuration of C12 to the chirality of C13, **3** was treated with NaOMe in methanol, and the obtained product triol **4** was converted to acetonide **5**. The conformation of the 2,2-dimethyl-1,3-dioxolane ring in **5** was analyzed by NOESY and HSQC experiments, both of which confirmed a *trans* relationship of H12 and H13 as evidenced by one-to-one NOE correlations between the acetonide methyl groups and oxymethine protons. Therefore, it was concluded that C13 has an *R*-configuration.

**Scheme 3:**
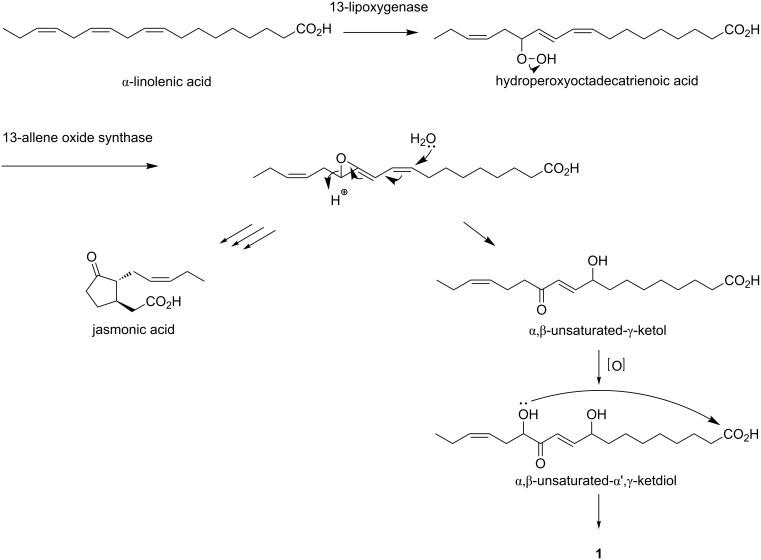
Plausible biosynthesis of sacrolide A (**1**).

Sacrolide A (**1**) is apparently a member of the oxylipins, lipid oxidation products reported from all organisms, and its biosynthesis should be routed through α-linolenic acid-derived allene oxide (Scheme 3) [16]. The addition of H_2_O to this intermediate gives α,β-unsaturated-γ-ketol, which accepts further oxidation at the α'-position, and macrolactonization of the resulting α',γ-ketodiol would yield **1**. Similar, but rather simpler metabolites were found from corn [17–18]. However, they comprise a pair of enantiomers and thus should be generated non-enzymatically from α-linoleic acid-derived allene oxide. In contrast, **1** is optically active and is more likely to be produced by a sequence of enzymatic reactions.

**Figure 3:**
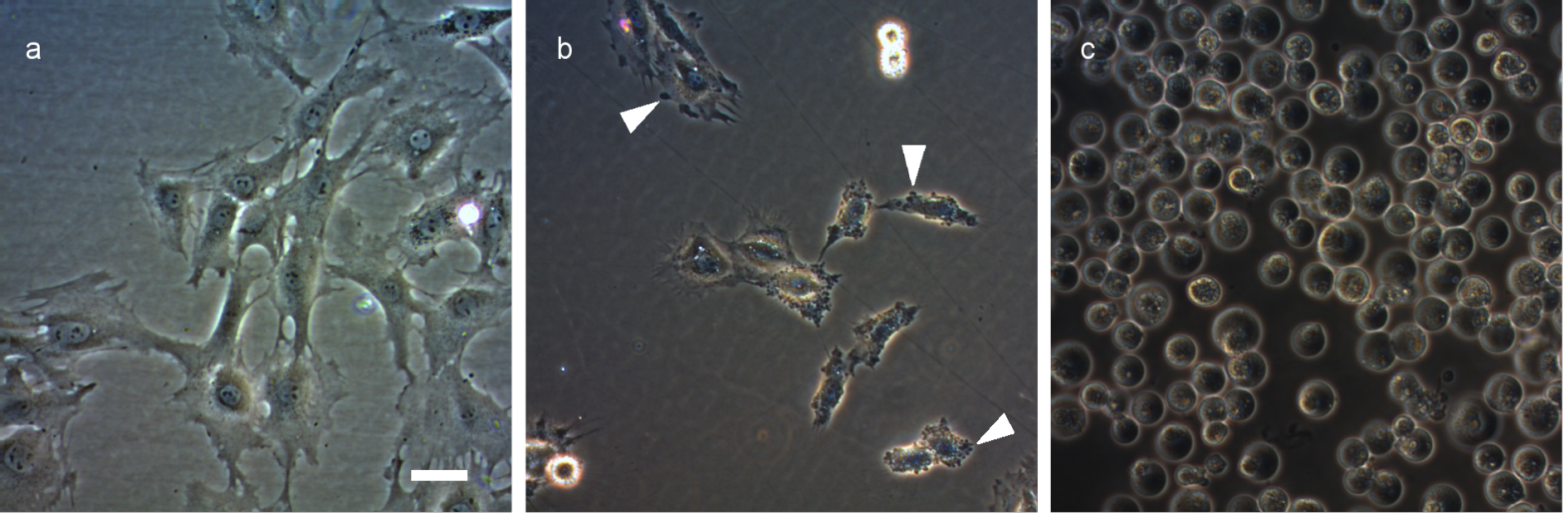
The effect of sacrolide A (**1**) on 3Y1 rat fibroblastic cells. (a) Control. (b) 45 min after exposure to 13 μM of **1**. (c) 20 h after exposure to 13 μM of **1**. Arrowheads indicate blebs on the cell surface. The magnification is identical in (a), (b) and (c), the bar represents 100 μm.

Sacrolide A (**1**) inhibited the growth of Gram-positive bacteria (*Micrococcus luteus*, *Streptomyces lividans*, *Staphylococcus aureus*, *Bacillus subtilis*), a yeast (*Saccharomyces cerevisiae*) and a fungus (*Penicillium chrysogenum*) (Table 2). Moreover, **1** was cytotoxic to 3Y1 rat fibroblasts (GI_50_ 4.5 μM). Under microscopic observation, cells exposed to 13 μM of **1** rapidly lost anchorage to the substratum. Blebs developed on the cellular surface (Figure 3B) and the cells eventually detached from the substratum and died (Figure 3C). Because this series of changes occurred within a time frame which was much shorter than the doubling time of the cells, **1** is is not expected to target cell cycle-associated events, such as the synthesis of proteins, DNA, or RNA. Despite their structural diversity, many oxylipins induce a common stress response in plants and animal cells, and this activity is attributed to their ability to modify the cysteinyl residues in proteins, thereby affecting redox-controlled signal transduction pathways [19]. The induction of membrane blebbing has been reported for lipophilic inhibitors of protein phosphatase 2A, which is a cysteine-dependent phosphatase [20–21]. However, **1** did not inhibit this enzyme (data not shown).

**Table 2:** Antimicrobial activity of sacrolide A (**1**).

*microbe*		MIC^a^ value (μg/mL)

Gram-positive	*Bacillus subtilis*	>8.0
	*Micrococcus luteus*	>8.0
	*Staphylococcus aureus*	0.5
	*Streptomyces lividans*	1.0

Gram-negative	*Escherichia coli*	>8.0

yeast	*Candida albicans*	8.0
	*Saccharomyces cerevisiae*	>8.0

fungus	*Penicillium chrysogenum*	1.0

^a^Minimum inhibitory concentration.

**Figure 4:**
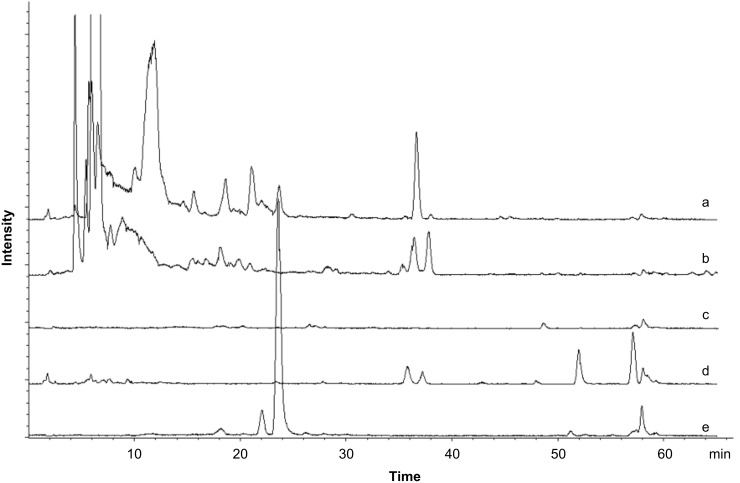
Extracted ion chromatograms for sacrolide A (**1**) molecular ion at *m/z* 307 [M – H]^−^ in the LC–MS analysis of aqueous 90% MeOH fractions from the ethanolic extracts of (a) raw alga, (b) dry product, (c) brined product, and (d) raw alga boiled in 3% NaHCO_3_ for 1 min. Chromatogram (e) shows the elution of purified sacrolide A (**1**) at *t*_R_ 23.6 min.

In Japanese cuisine, *A. sacrum* is used as an ingredient in soups and marinades or as a garnish for *sashimi* (thin sliced raw fish). It is sold dry or brined for nationwide distribution or as a fresh food item directly to local restaurants. Although a massive ingestion is rare to happen, the cytotoxicity of **1** raised concerns about potential food intoxication. To evaluate the presence of **1** in these products, extracts were prepared and examined by antifungal testing and LC–MS analysis. The products were softened or desalted by soaking in water and then subjected to the same extraction and fractionation scheme used at the initial antimicrobial screening. The aqueous 90% MeOH fraction of the raw sample exhibited significant antifungal activity (12 mm/6 mm Φ disk) against *Penicillium chrysogenum* at 200 μg/disk and an intense molecular ion of **1** at *m/z* 307 [M – H]^−^ in negative mode LC–MS analysis (Figure 4). In contrast, the same fractions from the commercial products were both inactive and devoid of a peak corresponding to **1**, which dispelled the concern about food intoxication for processed *A. sacrum*.

To eliminate the uncertainty with regard to the safety of eating substantial amounts of raw alga, a precooking treatment to manually deactivate **1** was desired. We hypothesized that the modification of the reactive enone functionality or the degradation of the cyclic structure may eliminate the bioactivity of **1,** and envisioned that the treatment with an alkaline solution may serve this purpose. In fact, by boiling the alga in 3% aqueous NaHCO_3_ for 1 min prior to extraction, the antifungal activity and a peak for **1** in the LC–MS analysis completely disappeared (Figure 4). Although the exact degradation pathway of **1** was not identified during this treatment, a facile deactivation of **1** in the raw alga was achieved in a fast, simple, and inexpensive manner, which offers a useful option when consumers wish to enjoy the crispy texture of fresh alga, but feel uneasy about the possibility of food intoxication.

## Conclusion

We explored a bioactive secondary metabolite from the very rare but edible cyanobacterium *A. sacrum* for the first time. *A. sacrum* is endemic to the Aso water system in Japan. As a result, we discovered a new oxylipin-derived macrolide **1** with a broad antimicrobial spectrum and cytotoxicity. Although the safety of this alga as food is secured by 250 years of consumption as a premium delicacy and the lack of a single episode of food intoxication, safety was reconfirmed by checking the absence of **1** in the commercial products. Moreover, a convenient precooking treatment was developed to deactivate **1** in the raw alga, which prevents food intoxication caused by a high-dose intake of **1** and allows for the consumption of a substantial amount of unprocessed alga.

In conclusion, the present study illustrates the importance of exploring untapped biological resources for the discovery of new bioactive molecules, and also encourages the careful conservation of a variety of habitats to maximize the availability of genetic resources.

## Experimental

### General methods

Cosmosil 75C18-PREP (Nacalai Tesque Inc., 75 µm) was used for ODS flash chromatography. NMR spectra were obtained by a Bruker AVANCE 500 spectrometer at 500 MHz for ^1^H. Residual solvent peaks at δ_H_/δ_C_ 7.27/77.0 ppm in CDCl_3_ were used as chemical shift reference signals. HRMS–ESI–TOF and LC–MS analyses were carried out by an Agilent 1200 HPLC-DAD system coupled with a Bruker micrOTOF mass spectrometer. Optical rotation and UV spectra were recorded on a JASCO DIP-3000 polarimeter and a Hitachi U-3210 spectrophotometer, respectively.

### Biological material

*A. sacrum* is commercially cultured by one of the authors (K. T.) in a groundwater-irrigated aquaculture pond located in Kumamoto, Kyushu District. The algal mass collected in a sieve basket was immediately frozen at −20 °C and shipped to the laboratory in October, 2012. Both the dry and brined *A. sacrum* products were purchased from Kisendo Ltd. (Fukuoka, Japan) in January 2014.

### Extraction and isolation

500 g of a water-thawed specimen of the alga was ground with an equal amount of Celite in EtOH (1 L). The resulting slurry was paper-filtered to separate an ethanolic extract and an algal cake, and the latter was extracted three more times with the same amount of alcohol. The combined extract (1.5 g) was partitioned between aqueous 60% MeOH (400 mL) and dichloromethane (400 mL × 3), the former of which was further separated between aqueous 90% MeOH and *n*-hexane, whereas the latter was further separated between H_2_O (200 mL) and *n*-BuOH (200 mL × 3). The most active aqueous MeOH layer (422.9 mg) was subjected to silica gel chromatography with a stepwise elution by *n*-hexane/EtOAc 4:1, 2:1, 1:1, 1:2, 1:4, chloroform/MeOH 99:1, 97:3, 95:5, 9:1, and chloroform/MeOH/H_2_O 6:4:1 to give ten fractions. The second fraction, the one with the highest activity, was gel-filtered on Sephadex LH-20 (chloroform/MeOH 1:1) followed by silica gel HPLC (Cosmosil 5SL-II 1 × 25 cm) eluted with *n*-hexane/Et_2_O (2:1) to yield **1** (6.0 mg). Starting with the second most active *n*-hexane layer (380.1 mg) and following the same procedure gave 1.5 mg of **1**.

Sacrolide A (**1**): yellow solid; soluble in MeOH, CHCl_3_, MeCN, EtOAc, and Et_2_O, and insoluble in H_2_O. [α]_D_^24.5^ +41.4 (*c* 0.115, MeCN); UV (MeCN) λ_max_, nm (ε) 232 (8000), 283 (320), 331 (160); HRMS–ESI (*m*/*z*): [M + Na]^+^ calcd for C_18_H_28_NaO_4_, 331.1880; found, 331.1889.

### Paper disk agar diffusion method

The antimicrobial potency of the chromatographic fractions was evaluated by a paper-disk agar diffusion method. Fractions at each stage of the purification were diluted to the same concentration with MeOH, and 10 μL aliquots were loaded on paper disks with 6 mm diameter, which were left until completely dried. A loop of the test organism, suspended in a small amount water, was mixed with liquefied agar medium precooled to nearly body temperature, and the inoculated medium was quickly poured into a sterile plastic dish. The medium was composed of 0.5% yeast extract, 1.0% tryptone, 1.0% NaCl, 0.5% glucose, and 1.5% agar. After the agar solidified, the drug-loaded disks were placed on the plate, and the test cultures were incubated at 32 °C for a day or two until the diameters of inhibitory haloes turned measurable.

### Microculture antimicrobial testing

100 μL of Mueller–Hinton broth supplemented with CaCl_2_·2H_2_O (5% w/v) and MgCl_2_·6H_2_O (2.5% w/v) were transferred to the wells of a sterilized 96 well microtiter plate. The test samples were dissolved in DMSO and diluted 20 times with the same broth medium. For a two-fold serial dilution along the columns each 100 μL aliquot of the test solution was added to the top row, mixed with the pre-dispensed medium, and the same amount was then transferred to the second row. The same operation was repeated until reaching the bottom row, where the last transfer was discarded to equalize the volume of the medium. The indication strains were grown on agar media (*Staphylococcus aureus* FDA209P JC-1, *Micrococcus luteus* ATCC9341, *Escherichia coli* NIHJ JC-2, *Saccharomyces cerevisiae* S100, and *Candida albicans* A9540) or in Sabouraud broth (*Penicillum chrysogenum* NBRC4626, *Streptomyces lividans* TK23) overnight and then diluted with sterile saline or the same broth to 0.5 McFarland (~10^8^ cfu/mL). Two μL of these suspensions were immediately inoculated to the microcultures, and the plates were incubated at 32 °C for 18–24 h. The concentration required to completely inhibit the growth of the microbes was defined as the minimum inhibitory concentration (MIC).

### Cytotoxicity testing and cell morphology observation

The cytotoxicity against 3Y1-B clone 1-6 rat fibroblasts and the action of sacrolide A (**1**) on the morphology of the same cell line were evaluated by the procedures described in [22].

**Method to precook raw alga for the elimination of sacrolide A (1):** A tablespoon of NaHCO_3_ (15 g) was added to a pot containing 500 mL of water, and the solution was boiled at high temperature. Then 40 g of raw *A. sacrum* was added and boiled with occasional stirring for 1 min. The boiled alga was drained in a colander, washed three times with cold water, and then subjected to the extraction and fractionation procedure described in the next section.

**Evaluation of the presence of sacrolide A (1) in two commercial products and precooked sample of *A. sacrum*:** Dry or brined alga was soaked in water for 30 min and weighed prior to extraction. A 20 g portion from each product, precooked or raw alga, was extracted with EtOH, and the extracts were separated by solvent partitioning into four fractions soluble in *n*-hexane, aqueous 90% MeOH, 1-BuOH, and water. The yields were: 33.2 mg, 12.0 mg, 12.7 mg, and 51.2 mg from dry alga; 10.9 mg, 8.1 mg, 9.8 mg, and 25.3 mg from brined alga; 10.6 mg, 5.0 mg, 16.4 mg, and 80.6 mg from precooked alga; and 19.6 mg, 17.8 mg, 12.7 mg, and 22.6 mg from raw alga. The fractions were subjected to antifungal susceptibility testing by using the paper-disk agar diffusion method and LC–MS analysis to evaluate the presence of **1**. Given a dose of 200 μg/disk none of the fractions from the commercial products or precooked sample exhibited an activity against *Penicillium chrysogenum*, whereas the aqueous 90% MeOH fraction from raw alga exhibited an inhibition circle with a diameter of 12.0 mm, which indicates the presence of **1**. The LC–MS analysis was conducted with a Cosmosil AR-II column (Nacalai Tesque, 2.0 mm × 150 mm) under the elution program of 40% MeCN for 5 min, 1%/min MeCN concentration ramped to 100%. The concentration was held for 10 min at a flow rate of 0.2 mL/min at 35 °C. Ten μL aliquots from 10 μg/mL DMSO solution of authentic **1** and 1 mg/mL DMSO solution of the aqueous 90% MeOH fraction from each algal sample were injected, and negative ESI was employed for mass spectral detection. The authentic **1** eluted at 23.6 min, showing a negative molecular ion at *m/z* 307. Based on this result, total ion chromatograms for the analytes were edited by extracting an *m/z* 307 ± 0.5 signal, which presented elution of **1** from the raw alga sample. In contrast, other samples were devoid of **1**.

## Supporting Information

File 1 Procedures for chemical conversion/derivatization, NMR assignments, copies of NMR, MS and UV spectra for **1**, NMR spectra for **2**, **3**, **3a** and **3b**.
