# Supplementary material for: Sacrolide A, a new antimicrobial and cytotoxic oxylipin macrolide from the edible cyanobacterium Aphanothece sacrum
Source: Beilstein J Org Chem. 2014 Aug 7;10:1808–16. doi: 10.3762/bjoc.10.190 (PMC4142850; doi:10.3762/bjoc.10.190)
Supplement: File 1 — Procedures for chemical conversion/derivatization, NMR assignments, copies of NMR, MS and UV spectra for 1, NMR spectra for 2, 3, 3a and 3b. [file Beilstein_J_Org_Chem-10-1808-s001.pdf]

Supporting Information

for

Sacrolide A, a new antimicrobial and cytotoxic oxylipin macrolide from the edible cyanobacterium *Aphanothece sacrum*

Naoya Oku[1], Miyako Matsumoto[1], Kohsuke Yonejima[1], Keijiroh Tansei[2], and Yasuhiro Igarashi*[1]

Address: [1] Biotechnology Research Center and Department of Biotechnology, Toyama Prefectural University, 5180 Kurokawa, Imizu, Toyama 939-0398, Japan and [2]Suizenjinori-Hompo Tanseidoh, 5-13-3 Tsuboi, Chuo-ku, Kumamoto, Kumamoto 860-0863, Japan

Email: Yasuhiro Igarashi* - yas@pu-toyama.ac.jp

*Corresponding author

Procedures for chemical conversion/derivatization, NMR assignments, copies of NMR, MS and UV spectra for 1, NMR spectra for 2, 3, 3a and 3b

Table of contents

Experimental Section.. S3

Sacrolide A (**1**)

^{1}H NMR spectrum ($CDCl_3$, 500 MHz) S6

^{13}C NMR spectrum ($CDCl_3$, 125 MHz) S7

COSY spectrum ($CDCl_3$, 500 MHz) S8

HSQC spectrum ($CDCl_3$, 500 MHz) S9

HMBC spectrum ($CDCl_3$, 500 MHz).. S10

HRESI mass spectrum ($CDCl_3$, 500 MHz) S11

UV spectrum ($CDCl_3$, 500 MHz) S12

(*R*)-12-Deoxo-12-hydroxysacrolide A (**3**)

^{1}H NMR spectrum ($CDCl_3$, 500 MHz) S13

COSY spectrum ($CDCl_3$, 500 MHz) S14

HSQC spectrum ($CDCl_3$, 500 MHz) S15

HMBC spectrum ($CDCl_3$, 500 MHz).. S16

bis-(*S*)-α-Methoxyphenylacetic acid esters **3a**

^{1}H NMR spectrum ($CDCl_3$, 500 MHz) S17

COSY spectrum ($CDCl_3$, 500 MHz) S18

HSQC spectrum ($CDCl_3$, 500 MHz) S19

HMBC spectrum ($CDCl_3$, 500 MHz).. S20

bis-(*R*)-α-Methoxyphenylacetic acid esters **3b**

^{1}H NMR spectrum ($CDCl_3$, 500 MHz) S21

COSY spectrum ($CDCl_3$, 500 MHz) S22

HSQC spectrum ($CDCl_3$, 500 MHz) S23

HMBC spectrum ($CDCl_3$, 500 MHz).. S24

Acetonide **5**

^{1}H NMR spectrum ($CDCl_3$, 500 MHz) S25

COSY spectrum ($CDCl_3$, 500 MHz) S26

HSQC spectrum ($CDCl_3$, 500 MHz) S27

HMBC spectrum ($CDCl_3$, 500 MHz).. S28

NOESY spectrum ($CDCl_3$, 500 MHz) S29

EXPERIMENTAL SECTION

Preparation of 9-*O*-acetyl sacrolide A (6)

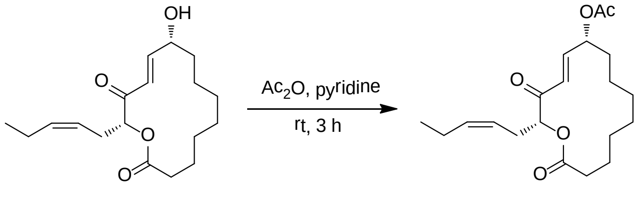

1 **6**

To the solution of sacrolide A (**6**: 2.0 mg, 0.0065 mmol) in pyridine (50 µL) was added acetic anhydride (50 µL) and the reaction mixture was stirred at an ambient temperature for 3 h. Removal of the reagent and solvent *in vacuo* yielded 9-*O*-acetyl sacrolide A (**6:** 2.2 mg). **6**; ^{1}H NMR (500 MHz, $CDCl_3$) δ_H 6.86 (1H, dd, *J*= 4.8, 15.8 Hz, H-10), 6.49 (1H, dd, *J*= 1.6, 16.1 Hz, H-11), 5.53 (1H, dd, *J*= 7.3, 10.8 Hz, H-16), 5.46 (1H, m, H-9), 5.29 (1H, dd, *J*= 7.4, 10.7 Hz, H-15), 5.25 (1H, t, *J*= 6.3 Hz, H-13), 2.57 (2H, t, *J*= 7.4 Hz, H-14), 2.49 (1H, m, H-2a), 2.38 (1H, m, H-2b), 2.08 (3H, s, CH_3CO-), 2.05 (2H, m, H-17), 1.80 (2H, m, H-8), 1.69 (2H, m, H-3), 1.45 (2H, m, H-6), 1.35 (2H, m, H-4), 1.31 (2H, m, H-5), 1.26 (2H, m, H-7), 0.96 (3H, t, *J*= 7.6 Hz, H-18); ^{13}C NMR (data assigned from HSQC, $CDCl_3$) δ_C 144.8 (C-10), 135.6 (C-16), 125.3 (C-11), 121.9 (C-15), 77.0 (C-13), 72.4 (C-9), 33.8 (C-2), 30.8 (C-8), 29.5 (C-7), 28.4 (C-14), 26.0 (2C, C-4, 6), 24.4 (C-3), 21.0 (3C, C-5, 17, *C*H_3CO-), 14.1 (C-18).

Preparation of (*R*)-12-deoxo-12-hydroxysacrolide A (3)

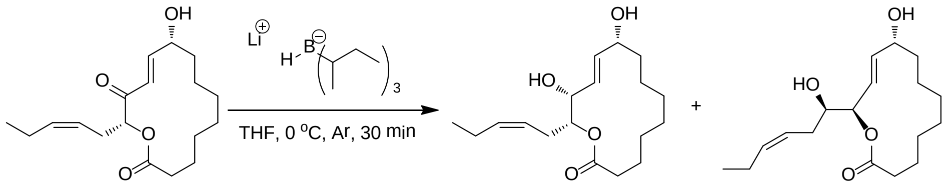

1 **3** **2**

To an ice-cooled solution of sacrolide A (**1**: 3.5 mg, 0.0114 mmol) in THF (150 µL) was added a solution of L-selectride (1 M in THF, 100µL) under Ar atmosphere, and the reaction was continued at an ambient temperature for 30 min with stirring. To quench the reaction, saturated aqueous NH_4Cl (150 µL) was added to the reaction mixture with cooling the vessel on ice. After stirring for 10 min, the reaction product was extracted with Et_2O (500 µL) for three times, and purified by ODS HPLC (column: Cosmosil AR-II, 1x25 cm, elution: linear gradient from 30 to 80% aqueous MeCN over 50 min, flow rate: 4 mL/min, monitored at 210 nm) to give (*R*)-12-deoxo-12-hydroxysacrolide A (**3**; 1.4 mg) along with its ester-exchanged isomer (**2**; 0.7 mg). **3**: ^{1}H NMR (500 MHz, $CDCl_3$) δ_H 5.77 (1H, dd, *J*= 1.9, 7.9, 15.4 Hz, H-10), 5.65 (1H, dd, *J*= 3.2, 15.8 Hz, H-11), 5.55 (1H, dd, *J*= 7.3, 10.7 Hz, H-16), 5.34 (1H, dd, *J*= 7.5, 10.7 Hz, H-15), 4.88 (1H, m, H-13), 4.33 (1H, m, H-12), 4.09 (1H, t, *J*= 9.2 Hz, H-9), 2.52 (2H, m, H-14), 2.39 (1H, m, H-2a), 2.30 (1H, m, H-2b), 2,12 (2H, m, H-17), 1.69 (2H, m, H-8), 1.59 (2H, m, H-3), 1.44 (2H, m, H-7), 1.32 (2H, m, H-4), 1.24 (2H, m, H-6), 1.15 (2H, m, H-5), 0.98 (3H, t, *J*= 7.3 Hz, H-18); ^{13}C NMR (125 MHz, $CDCl_3$) δ_C 173.2 (C-1), 135.6 (C-16), 133.7 (C-10), 132.4 (C-11), 122.9 (C-15), 75.3 (C-13), 73.2 (C-9), 70.8 (C-12), 35.4 (2C, C-7, 8), 34.5 (C-2), 28.6 (C-14), 25.6 (C-4), 23.8 (C-3), 21.8 (2C, C-5, 6), 20.6 (C-17), 14.3 (C-18). **2**: ^{1}H NMR (500 MHz, $CDCl_3$) δ_H 5.86 (1H, dd, *J*= 7.6, 15.7 Hz, H-10), 5.72 (1H, dd, *J*= 8.6, 15.7 Hz, H-11), 5.59 (1H, td, *J*= 7.3, 10.7 Hz, H-16), 5.41 (1H, td, *J*= 7.7, 10.4 Hz, H-15), 5.30 (1H, dd, *J*= 5.8, 8.5 Hz, H-12), 4.15 (1H, m, H-9), 3.76 (1H, td, *J*= 7.0, 5.8 Hz, H-13), 2.41 (1H, ddd, *J*= 3.0, 8.2, 12.7 Hz, H-2a), 2.29 (2H, brt, *J*= 7.0 Hz, H-14), 2.22 (1H, *J*= 3.2, 9.8, 12.9 Hz, H-2b), 2,06 (2H, qd, *J*=7.5, 7.4 Hz, H-17), 1.70 (1H, m, H-8a), 1.63 (1H, m, H-3a), 1.59 (1H, m, H-3b), 1.52 (1H, m, H-8b), 1.36 (1H, m, H-7a), 1.31, 1.29,

1.22 (6H, H-4, -5, -6), 1.17 (1H, m, H-7b), 0.98 (3H, t, *J*= 7.5 Hz, H-18); ^{13}C NMR (125 MHz, $CDCl_3$) δ_C 173.2 (C-1), 139.7 (C-10), 135.4 (C-16), 126.7 (C-11), 123.3 (C-15), 76.3 (C-12), 72.9 (C-9), 72.2 (C-13), 35.0 (2C, C-2, -8), 31.0 (C-14), 26.3, 25.3, 25.2 (C-4, -5, -6), 24.4 (C-3), 21.7 (C-7), 20.7 (C-17), 14.1 (C-18).

Preparation of *bis*-(*S*)- or (*R*)-α-methoxyphenylacetic acid esters 3a and 3b

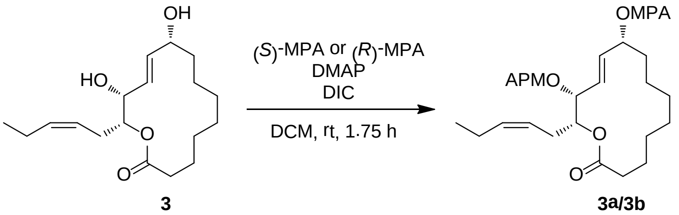

To a solution of **3** (1.6 mg, 5.15 μmol) in dichloromethane (50 μL) was added a one-third micro spatula of (*S*)-α-methoxyphenylacetic acid (MPA) and ten equivalent of *N,N'*-diisopropylcarbodiimide (8 μL, 54.5 μmol), and the reaction was continued for 100 min at an ambient temperature. To this solution was added ice-cooled 1N HCl (150 μL), and after stirring for 5 min, the reaction mixture was extracted with EtOAc (150 μL) for three times. The combined extract was passed through an aminopropyl-modified silica gel column made in a Pasteur pipet, and the eluted substance was purified by ODS HPLC (column: Cosmosil AR-II, 1x25 cm, solvent: 80% aqueous MeCN, flow rate: 4 mL/min, monitored at 210 nm) to give 12-hydroxysacrolide bis-(*S*)-MPA ester (**3a**; 1.4 mg). **3a:** ^{1}H NMR (500 MHz, $CDCl_3$) δ_H 5.54 (1H, m, H-12), 5.51 (1H, dd, *J*= 7.0, 10.8 Hz, H-16), 5.45 (1H, dd, *J*= 4.2, 16.0 Hz, H-11), 5.24 (1H, dd, *J*= 7.5, 10.8 Hz, H-15), 5.01 (1H, dt, *J*= 2.8, 7.8, 14.4 Hz, H-9), 4.96 (1H, dt, *J*= 1.6, 7.2, 12.6 Hz, H-13), 4.72 (1H, dd, *J*= 1.9, 15.7 Hz, H-10), 2.36 (3H, m, H-2a, 14), 2.27 (1H, m, H-2b), 2.01 (2H, m, H-17), 1.53 (2H, m, H-3), 1.44 (2H, m, H-4), 1.25 (2H, m, H-6), 1.19 (2H, m, H-5), 1.07 (2H, m, H-7), 1.03 (2H, m, H-8), 0.96 (3H, t, *J*= 7.5 Hz, H-18); ^{13}C NMR (125 MHz, $CDCl_3$) δ_C 169.5 (C-1), 135.6 (C-16), 129.7 (C-10), 128.7 (C-11), 122.1 (C-15), 74.6 (C-9), 73.1 (C-13), 72.3 (C-12), 34.1 (C-2), 31.3 (3C, C-4, 6, 7), 28.7 (C-14), 25.3 (C-5), 23.7 (C-3), 20.6 (C-8), 20.5 (C-17), 14.4 (C-18).

The same procedure with (*R*)-MPA yielded 1.4 mg of 12-hydroxysacrolide bis-(*R*)-MPA ester **3b** from 0.8 mg of the starting material **3**. **3b:** ^{1}H NMR (500 MHz, $CDCl_3$) δ_H 5.45 (3H, m, H-10, 11, 12), 5.35 (1H, dd, *J*= 7.2, 10.8 Hz, H-16), 5.23 (1H, m, H-9), 4.97 (1H, dd, *J*= 7.6, 10.7 Hz, H-15), 4.80 (1H, m, H-13), 2.35 (1H, m, H-2a), 2.25 (1H, m, H-2b), 1.97 (2H, m, H-14), 1.71 (4H, m, H-4, 17), 1.58 (4H, m, H-3, 8), 1.33 (2H, m, H-5), 1.29 (2H, m, H-6), 1.15 (2H, m, H-7), 0.84 (3H, t, *J*= 7.6, H-18); ^{13}C NMR (125 MHz, $CDCl_3$) δ_C 172.8 (C-1), 135.4 (C-16), 130.4 (C-10), 127.9 (C-11), 122.2 (C-15), 74.5 (C-9), 73.4 (C-13), 72.5 (C-12), 31.8 (C-8), 34.2 (C-2), 27.8 (C-14), 25.4 (C-5), 23.5 (C-3), 21.0 (2C, C-6, 7), 20.4 (2C, C-4, 17), 14.2 (C-18).

Preparation of acetonide 5 via ring-opened derivative 4

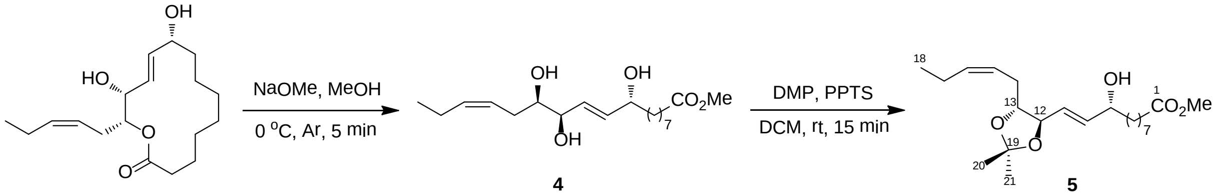

To an ice-cooled solution of **3** (0.6 mg, 0.00193 mmol) in MeOH (100 μL) was added 0.5 M NaOMe (50 μL, 0.025 mmol), and the reaction mixture was stirred for 5 min under Ar atmosphere. To quench the excess reagent, aqueous NH_4Cl (1 mL: 0.075 mmol) was added, and after stirring the reaction for 5 min, MeOH was removed under a stream of Ar. The water suspension was extracted three times with Et_2O (500 μL) to give methyl ester **4** (0.3 mg).

To compound **4** (0.7 mg, 0.00204 mmol) dissolved in dichloromethane (180 μL) was added 2,2-dimethoxypropane (DMP, 180 μL) and pyridinium *p*-toluenesulfonate (PPTS, 2.0 mg), and the reaction mixture was stirred for 15 min at an ambient temperature. After removing the solvent and DMP *in vacuo*, the resulting residue was suspended in H_2O (500 μL) and then extracted with Et_2O (500 μL×3). Purification of the organic extract by HPLC (column: Cosmosil AR-II, 1x25 cm, elution: linear gradient from 30 to 80% aqueous MeCN over 50 min, flow rate: 4 mL/min, monitored at 210 nm) yielded acetonide **5** (0.4 mg). **5**; ^{1}H NMR (500 MHz, $CDCl_3$) δ_H 5.85 (1H, dd, *J*=5.8, 15.4 Hz, H-10), 5.67 (1H, dd, *J*=0.6, 7.5, 15.4 Hz, H-11), 5.52 (1H, m, H-16), 5.40 (1H, *J*=7.6, 10.7 Hz, H-15), 4.16 (1H, m, H-9), 4.07 (1H, t, *J*=7.9 Hz, H-12), 3.75 (1H, td, *J*=5.7, 8.2 Hz, H-13), 3.68 (3H, s, 1-CH_3), 2.36 (2H, m, H-14), 2.31 (2H, t, *J*= 7.6 Hz, H-2), 2.06 (2H, qui, *J*=7.6 Hz, H-17), 1.62 (2H, m, H-3), 1.52 (2H, m, H-8), 1.43 (3H, s, H-21), 1.42 (3H, s, H-20), 1.31 (6H, m, H-5, 7, 8), 0.98 (3H, t, *J*= 7.6 Hz, H-20); ^{13}C NMR (125 MHz, $CDCl_3$) δ_C 174.4 (C-1), 138.4 (C-11), 137.8 (C-10), 134.2 (C-16), 123.2 (C-15), 108.5 (C-19), 81.2 (C-12), 80.3 (C-13), 71.8 (C-9), 51.4 (1-OCH_3), 37.0 (C-8), 34.0 (C-2), 29.4 (C-14), 29.1 (C-4, -5, -6 ,-7), 27.0 (2C, C-20, -21), 24.9 (C-3), 20.6 (C-17), 14.1 (C-18).

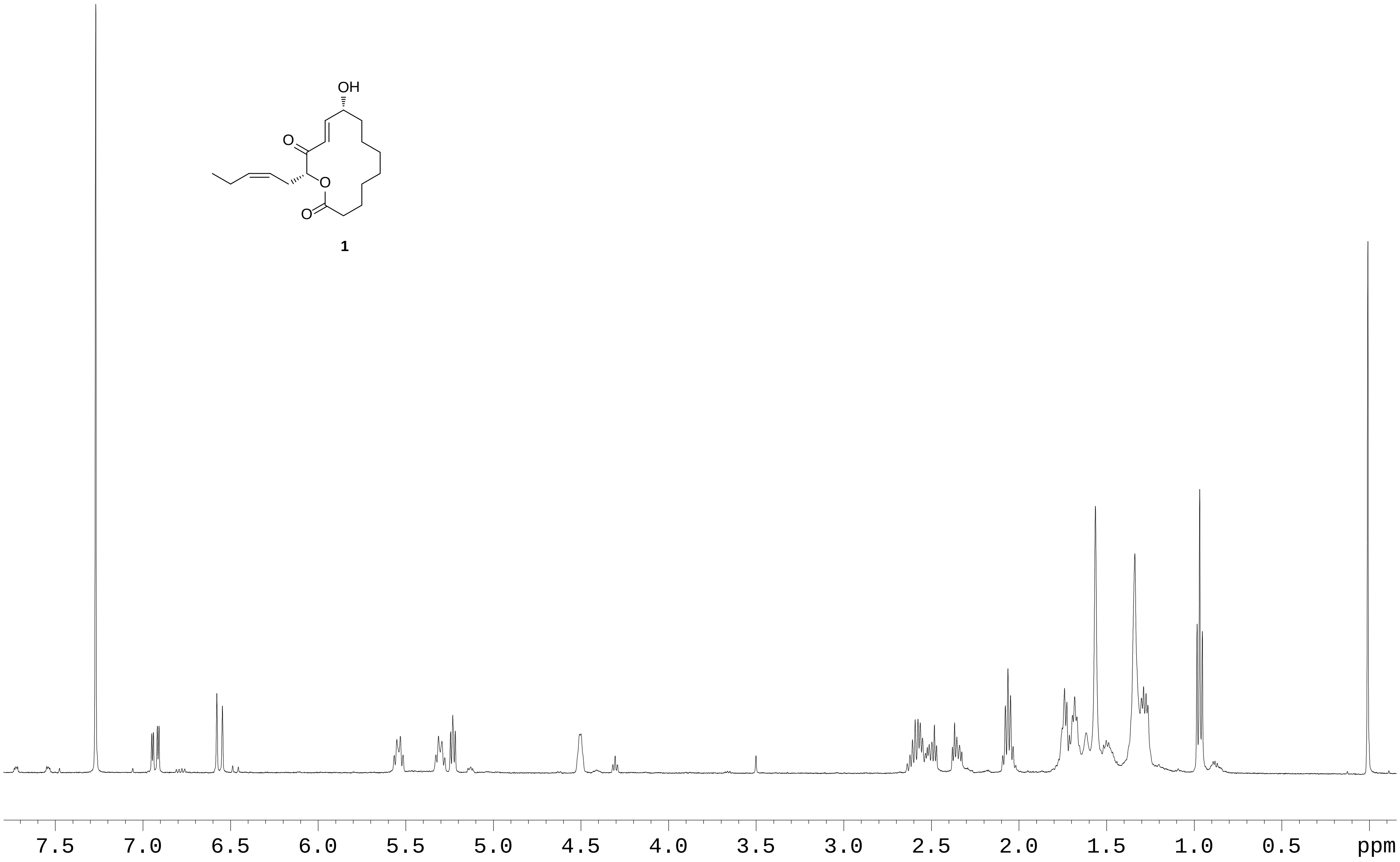

^{1}H NMR spectrum of sacrolide A (**1**) (500 MHz, $CDCl_3$).

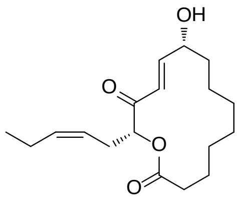

1

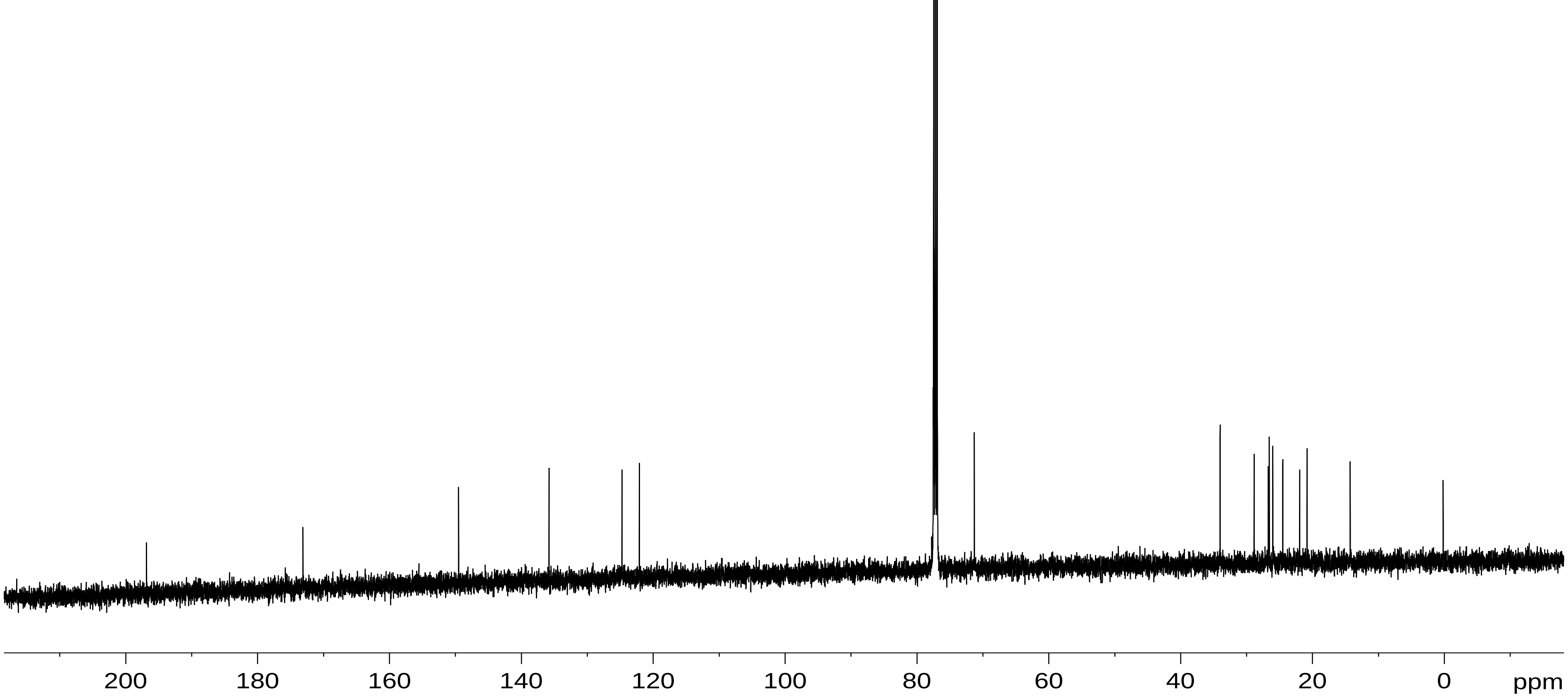

^{13}C NMR spectrum of sacrolide A (**1**) (125 MHz, $CDCl_3$).

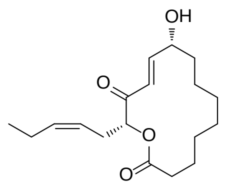

1

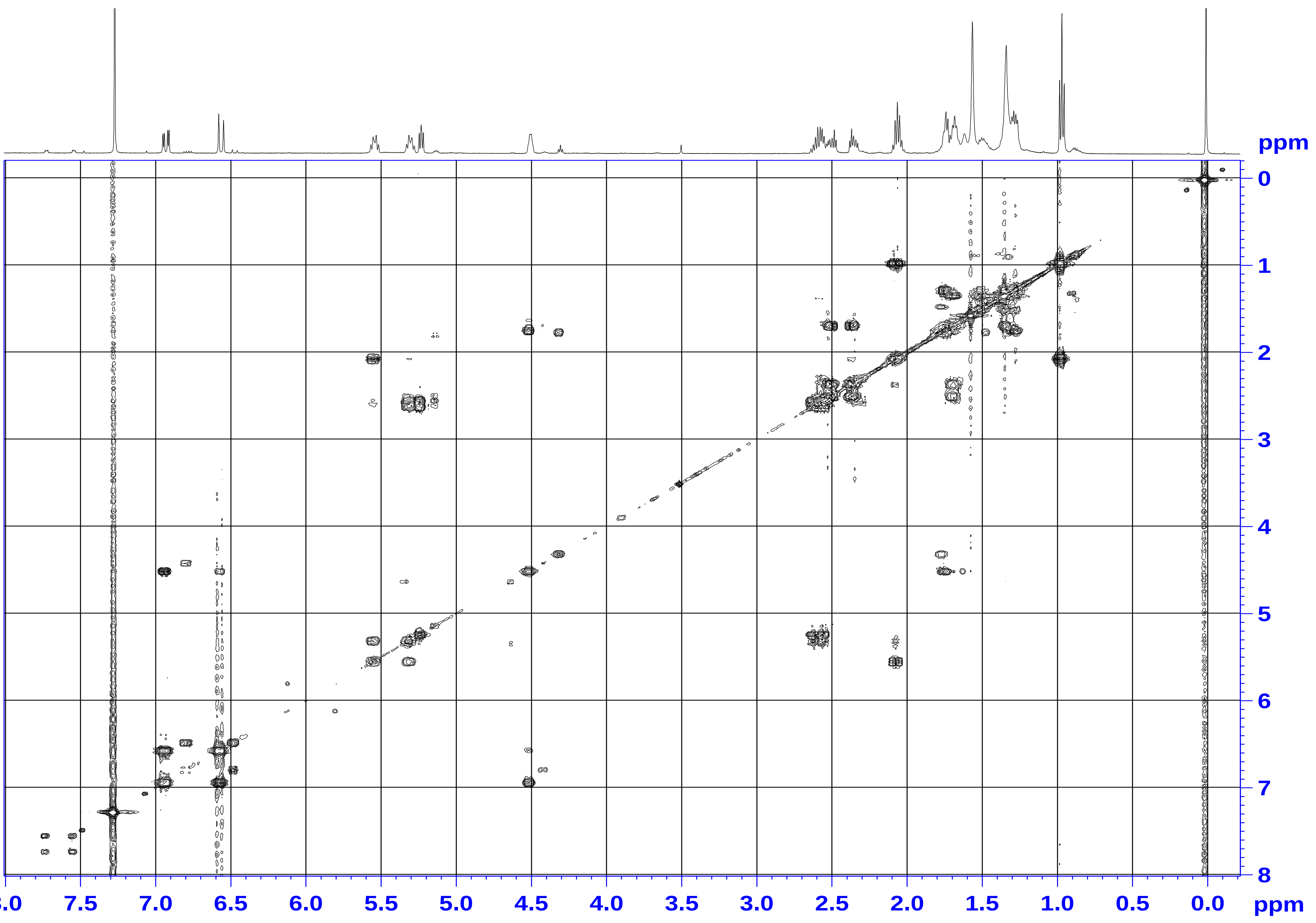

COSY spectrum of sacrolide A (**1**) (500 MHz, $CDCl_3$).

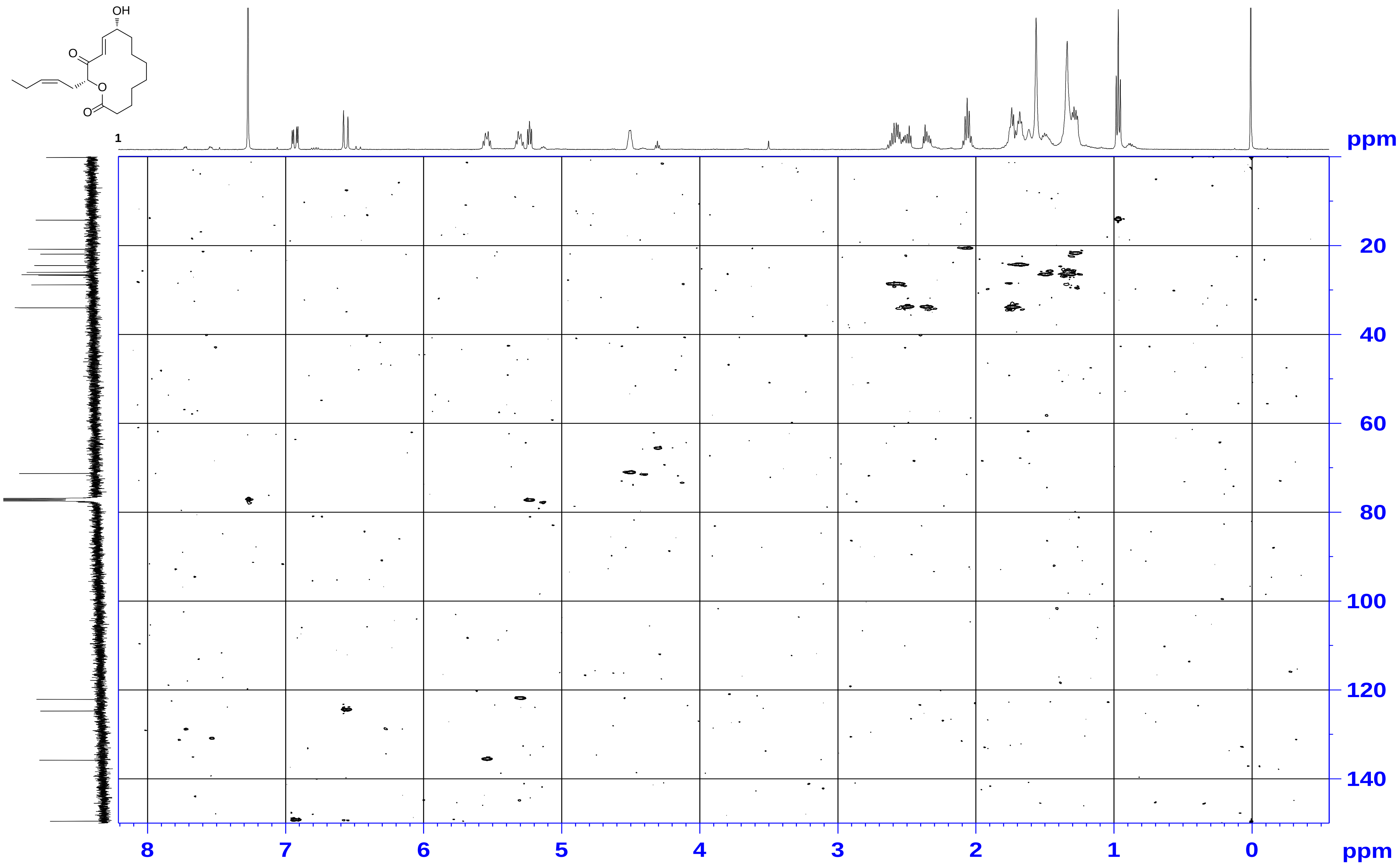

HSQC spectrum of sacrolide A (**1**) (500 MHz, $CDCl_3$).

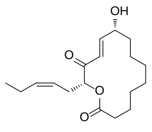

1

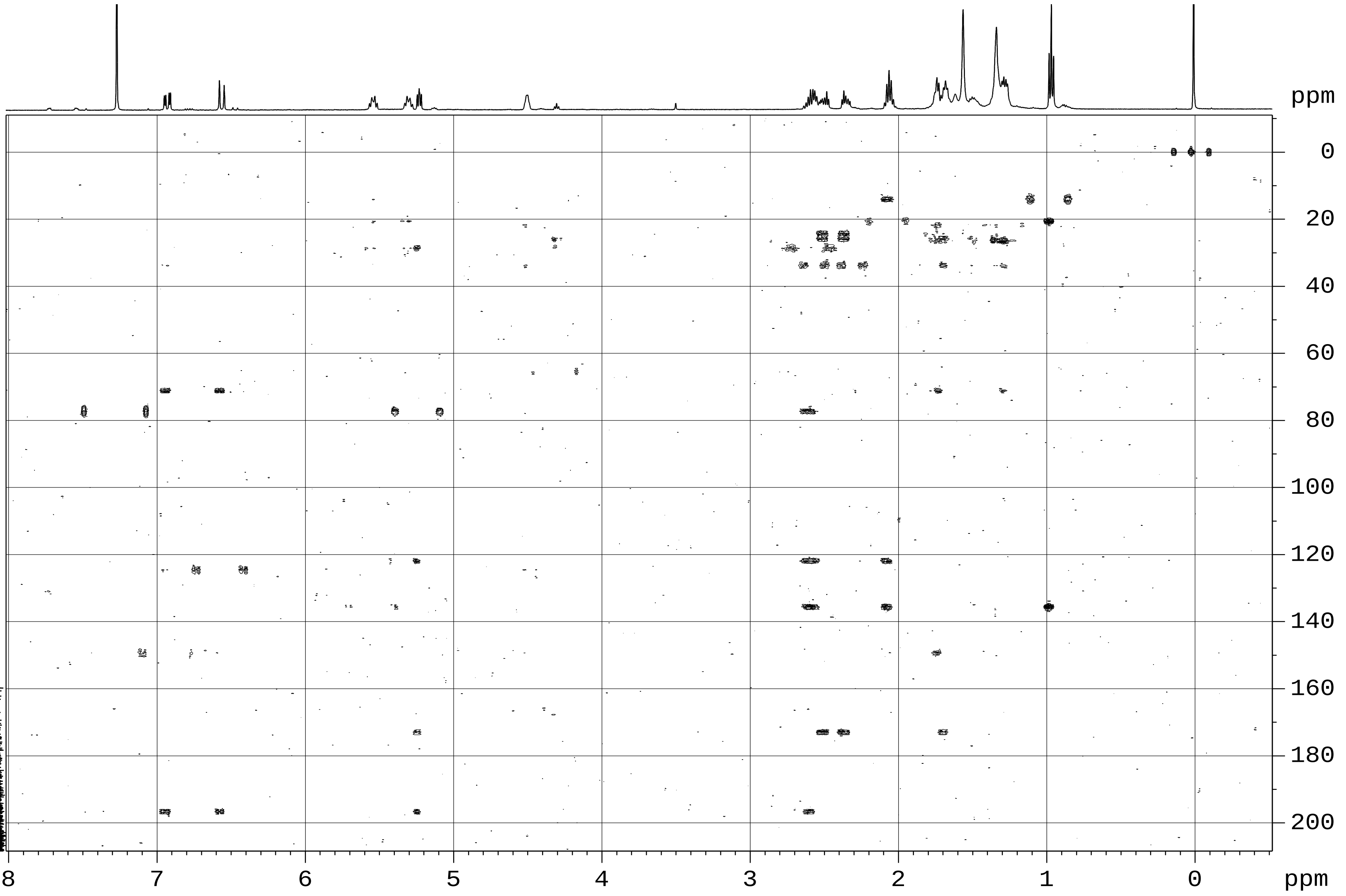

HMBC spectrum of sacrolide A (**1**) (500 MHz, $CDCl_3$).

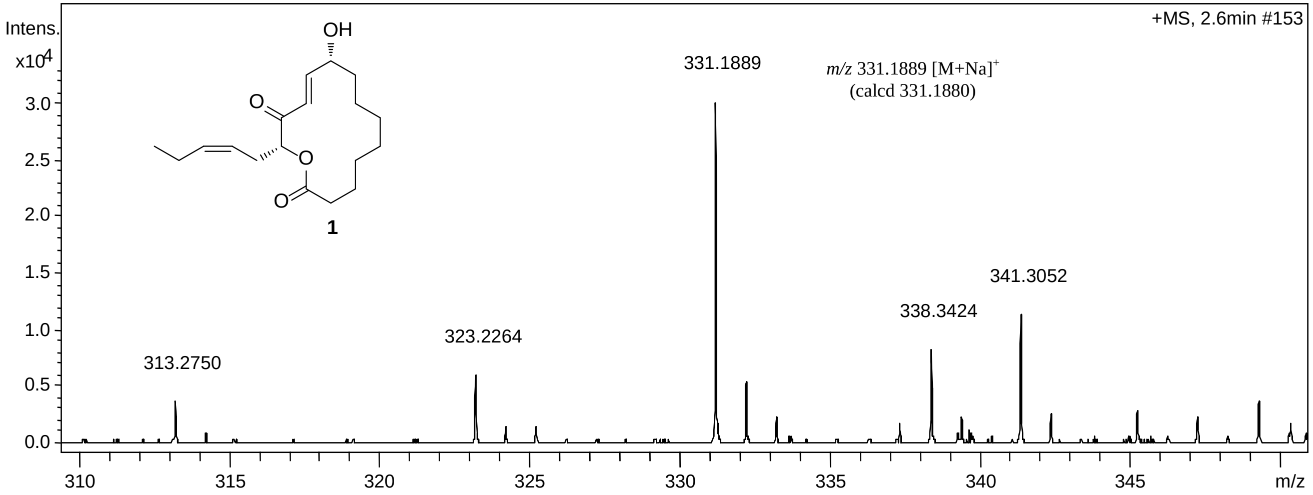

HRESI mass spectrum of sacrolide A (**1**)

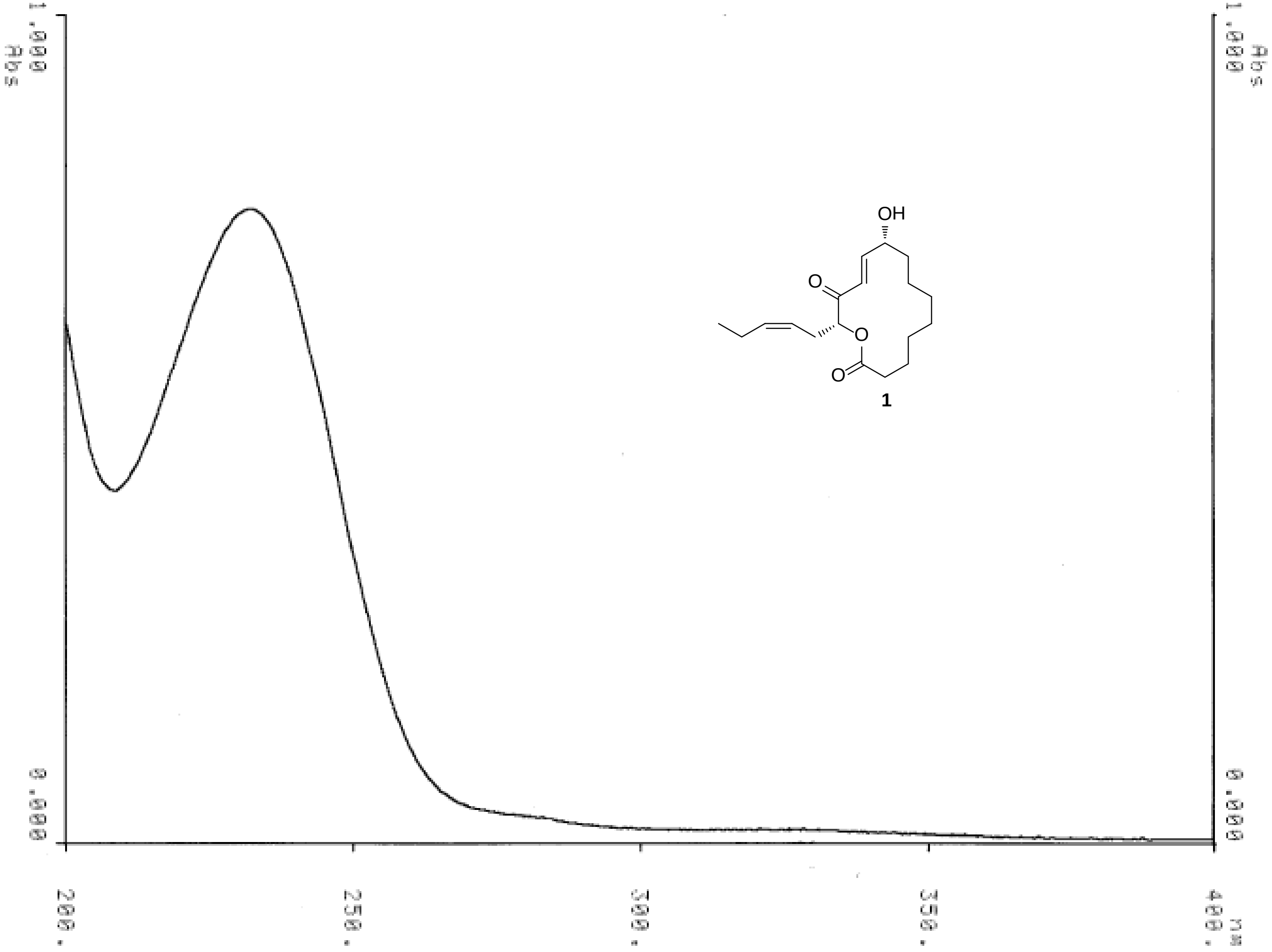

UV spectrum of sacrolide A (**1**) (30 µg/mL, MeCN).

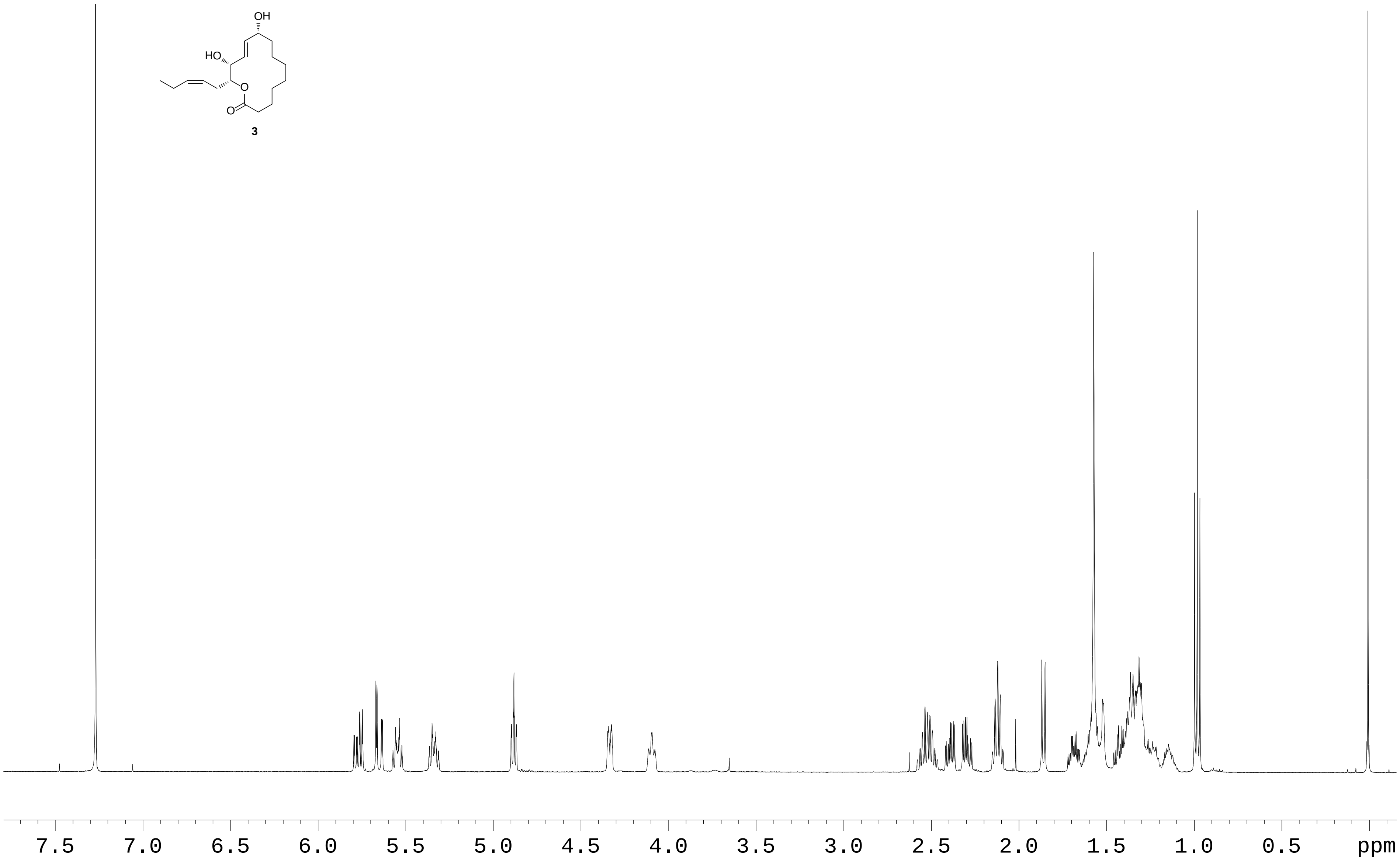

^{1}H NMR spectrum of (*R*)-12-hydroxysacrolide A (**3**) (500 MHz, $CDCl_3$).

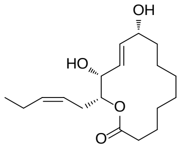

3

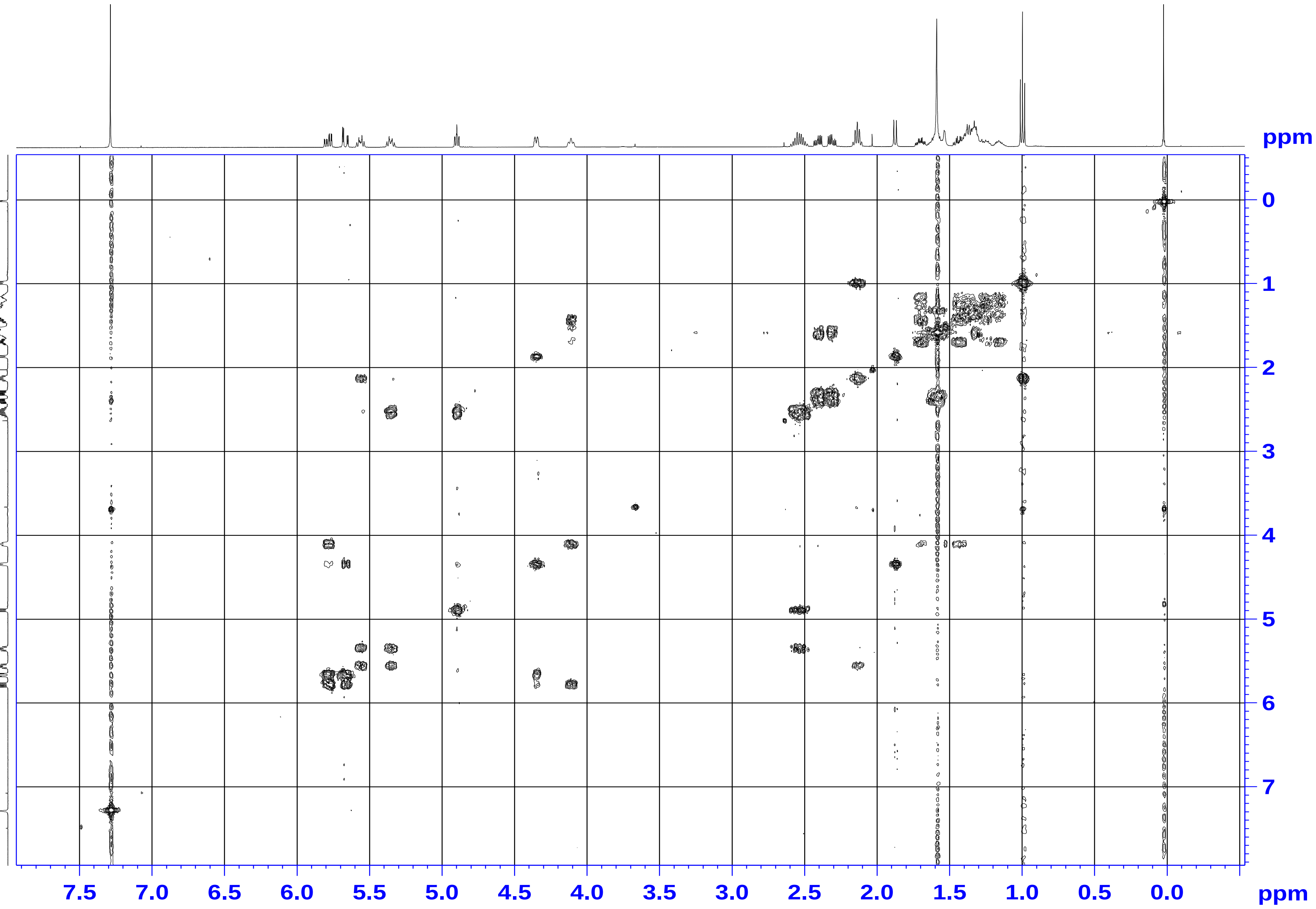

COSY spectrum of (*R*)-12-hydroxysacrolide A (**3**) (500 MHz, $CDCl_3$).

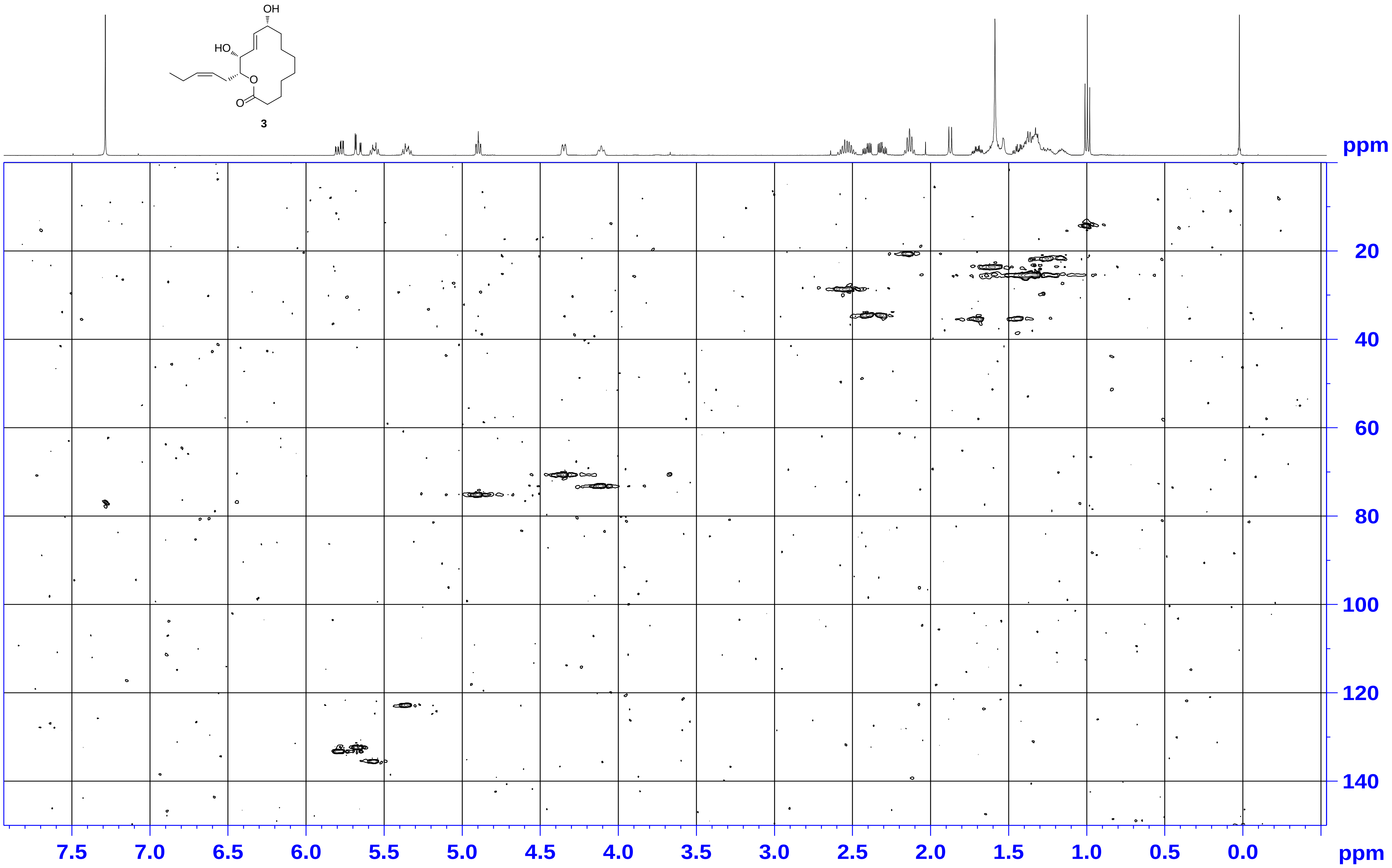

HSQC spectrum of (*R*)-12-hydroxysacrolide A (**3**) (500 MHz, $CDCl_3$).

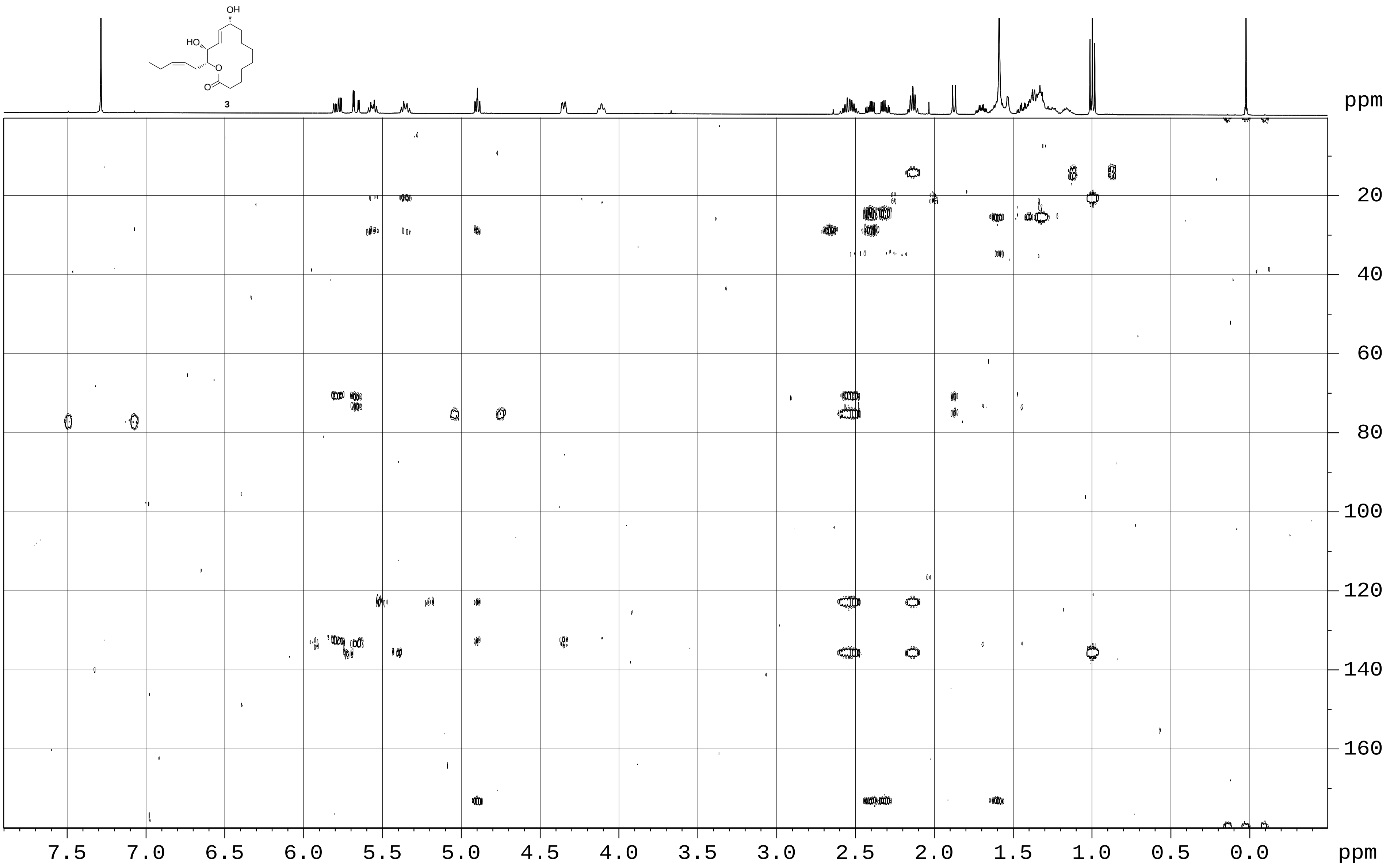

HMBC spectrum of (*R*)-12-hydroxysacrolide A (**3**) (500 MHz, $CDCl_3$).

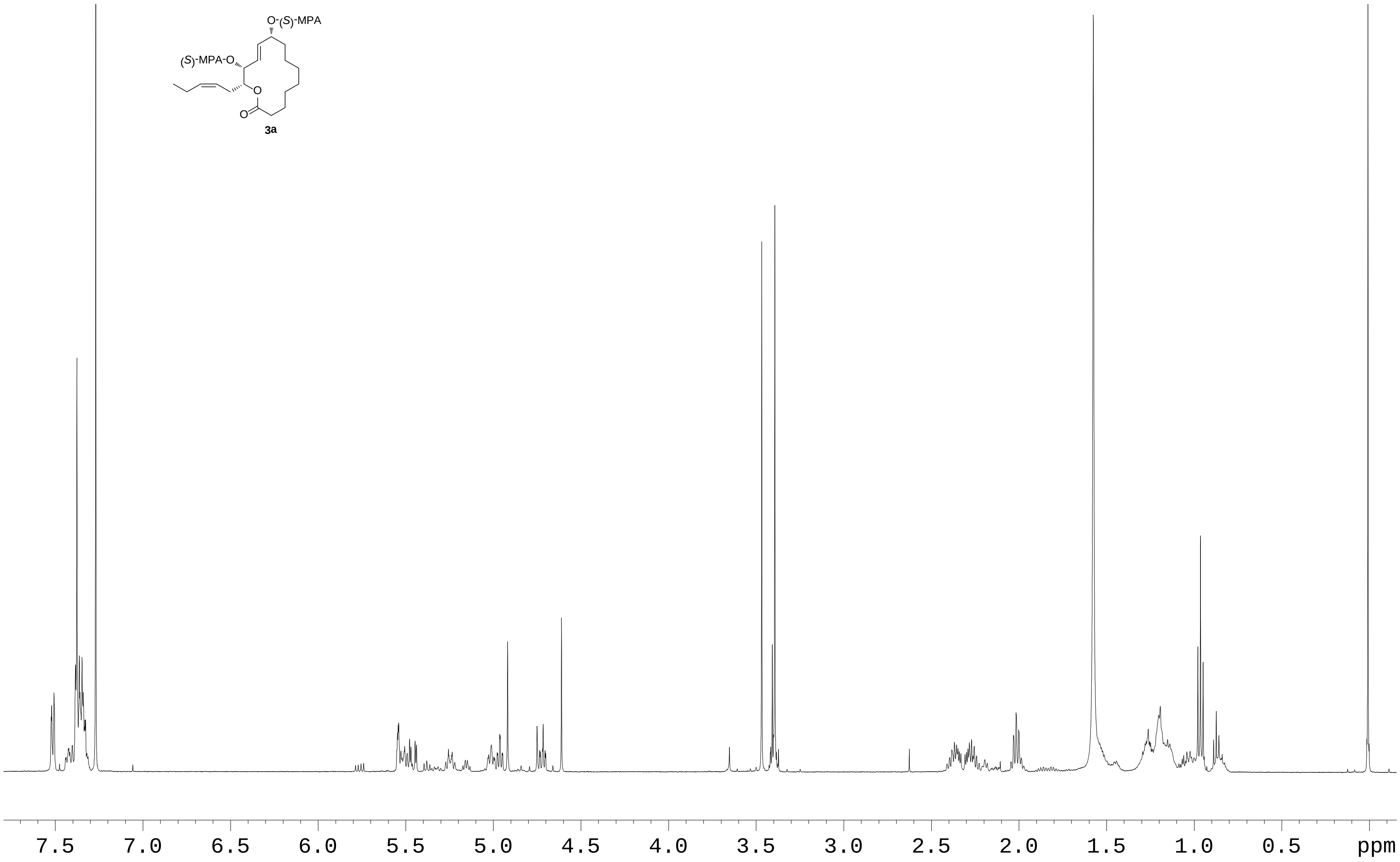

^{1}H NMR spectrum of *bis*-(*S*)-α-methoxyphenylacetic acid esters **3a** (500 MHz, $CDCl_3$).

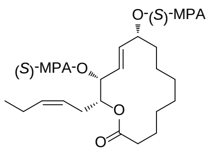

3a

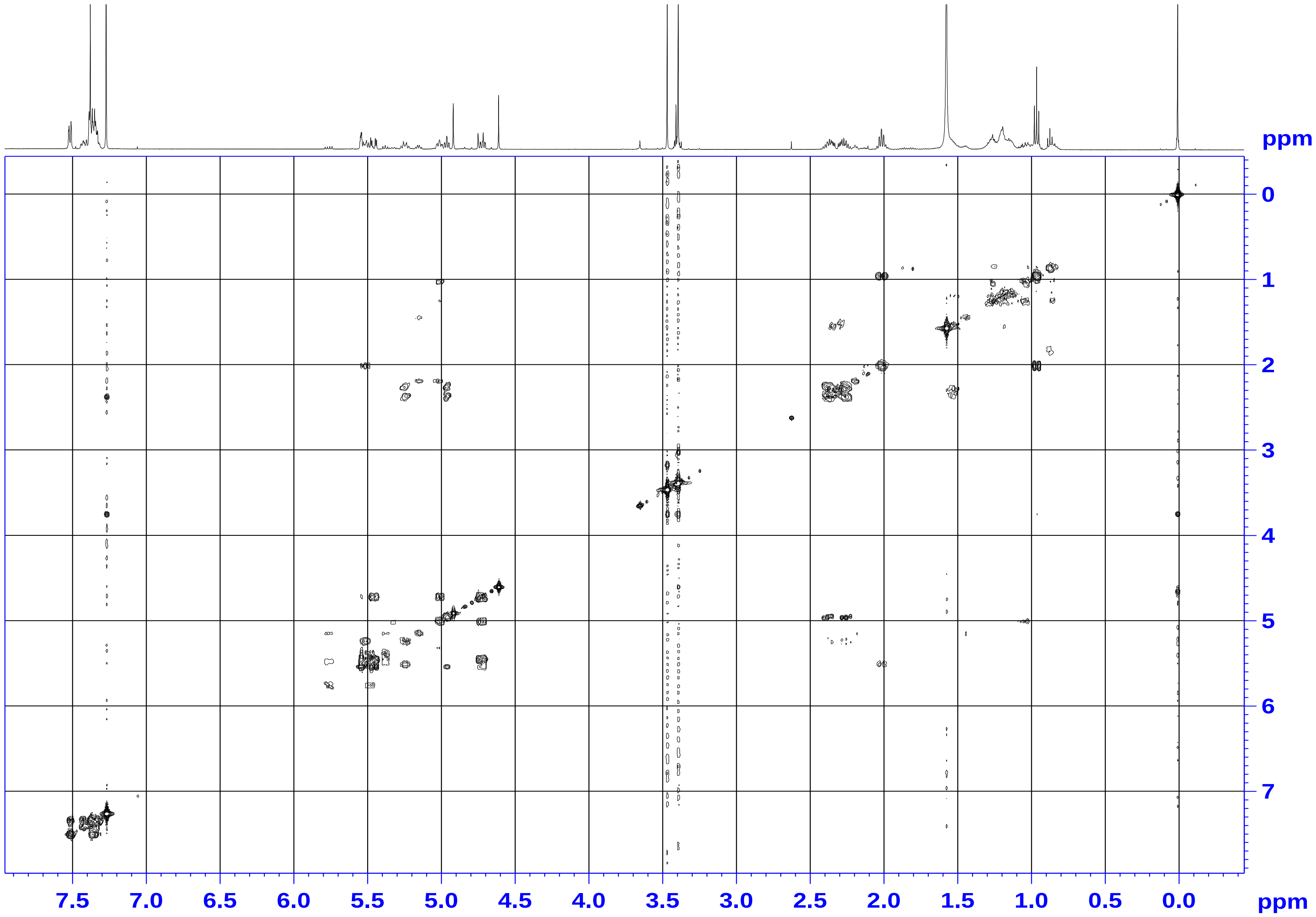

COSY spectrum of *bis*-(*S*)-α-methoxyphenylacetic acid esters **3a** (500 MHz, $CDCl_3$).

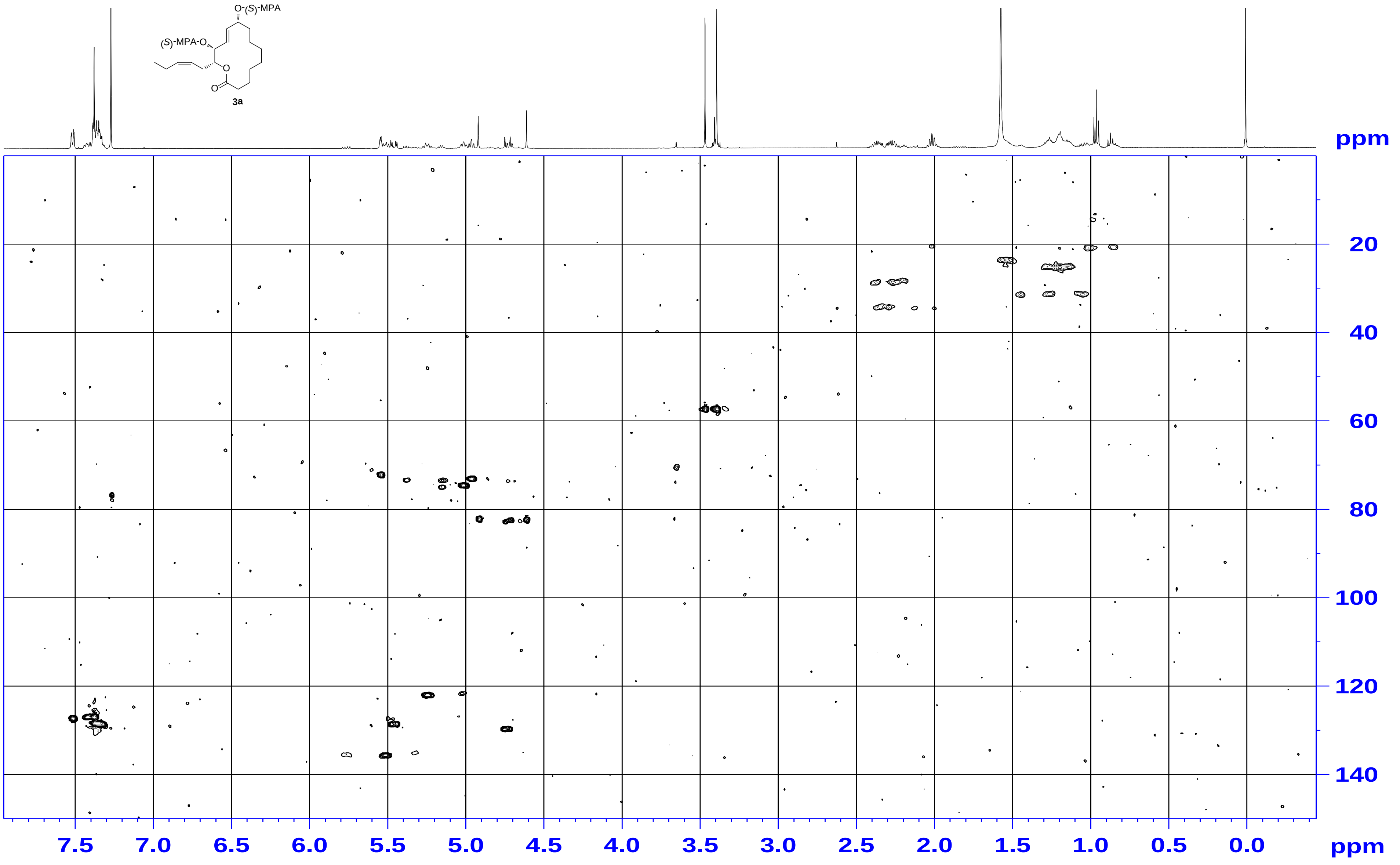

HSQC spectrum of *bis*-(*S*)-α-methoxyphenylacetic acid esters **3a** (500 MHz, $CDCl_3$).

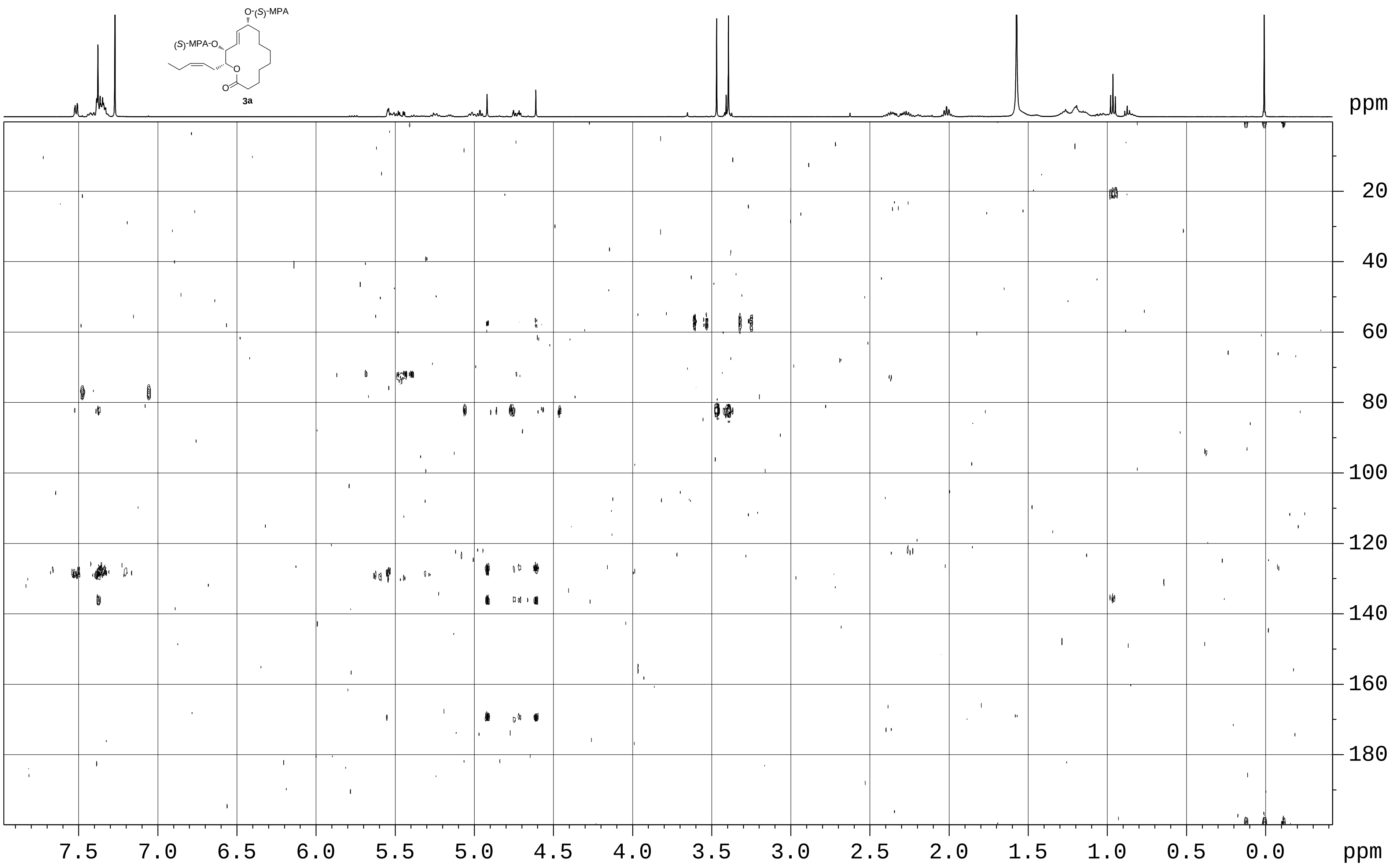

HMBC spectrum of *bis*-(*S*)-α-methoxyphenylacetic acid esters **3a** (500 MHz, $CDCl_3$).

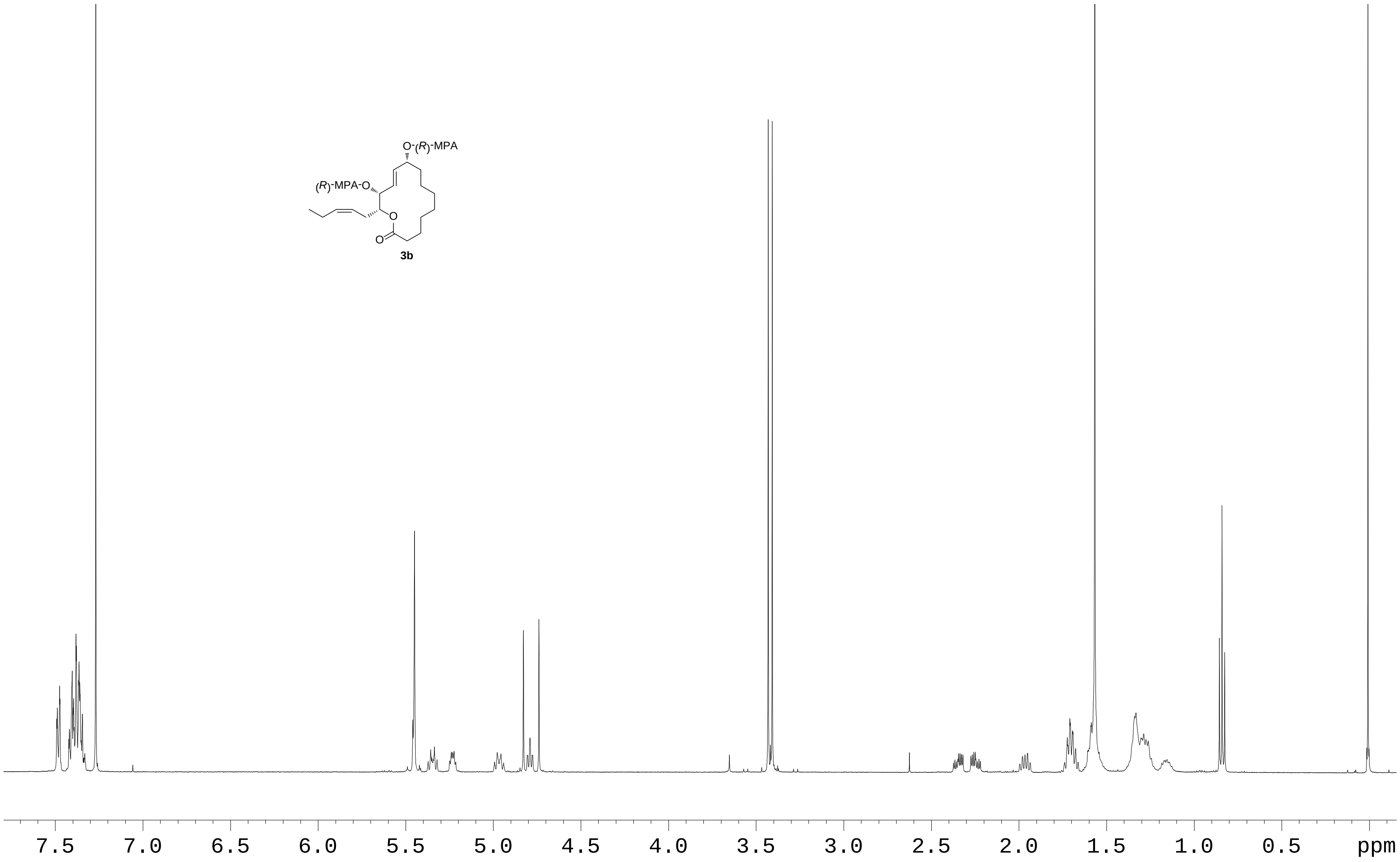

^{1}H NMR spectrum of *bis*-(*R*)-α-methoxyphenylacetic acid esters **3b** (500 MHz, $CDCl_3$).

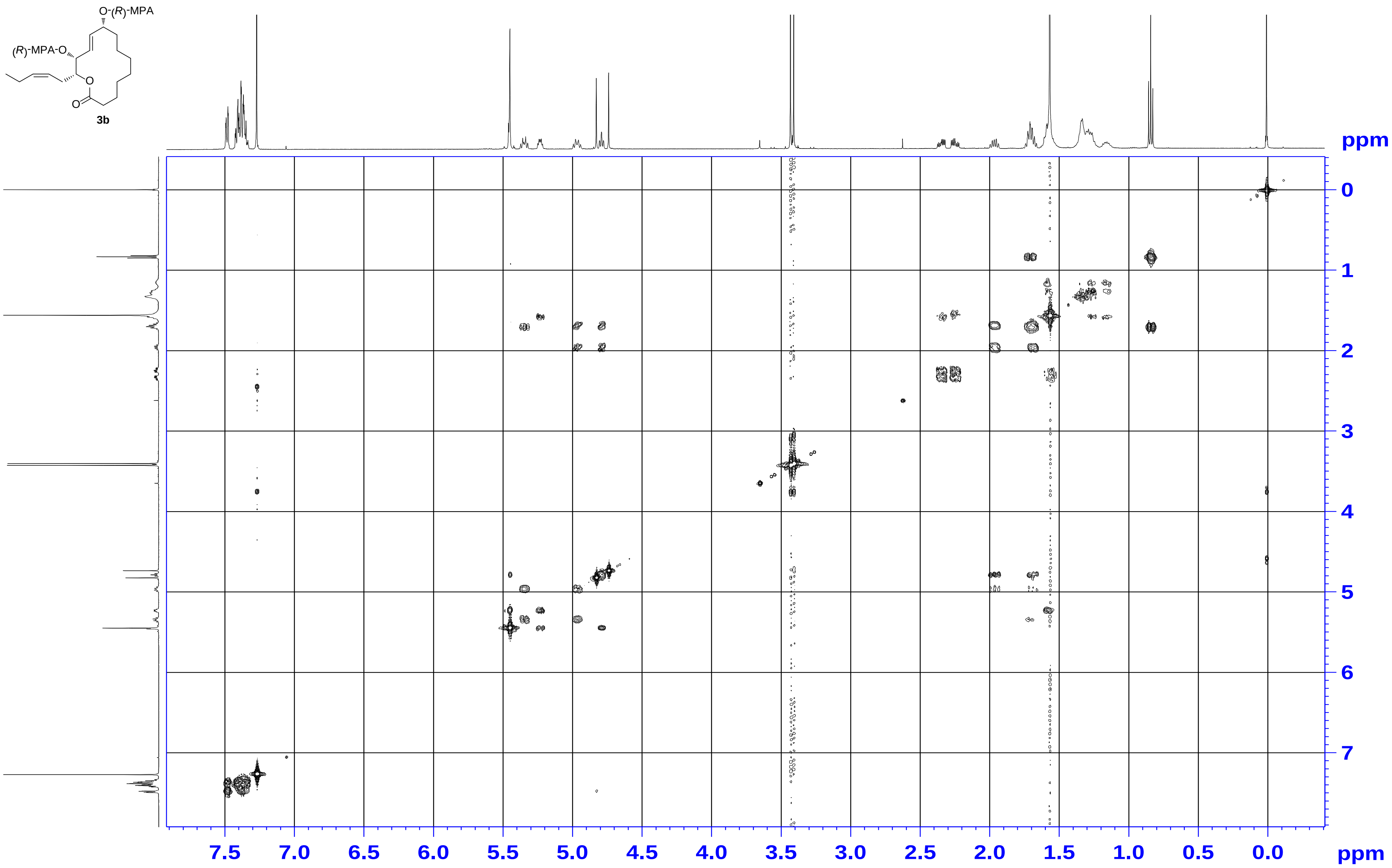

COSY spectrum of *bis*-(*R*)-α-methoxyphenylacetic acid esters **3b** (500 MHz, $CDCl_3$).

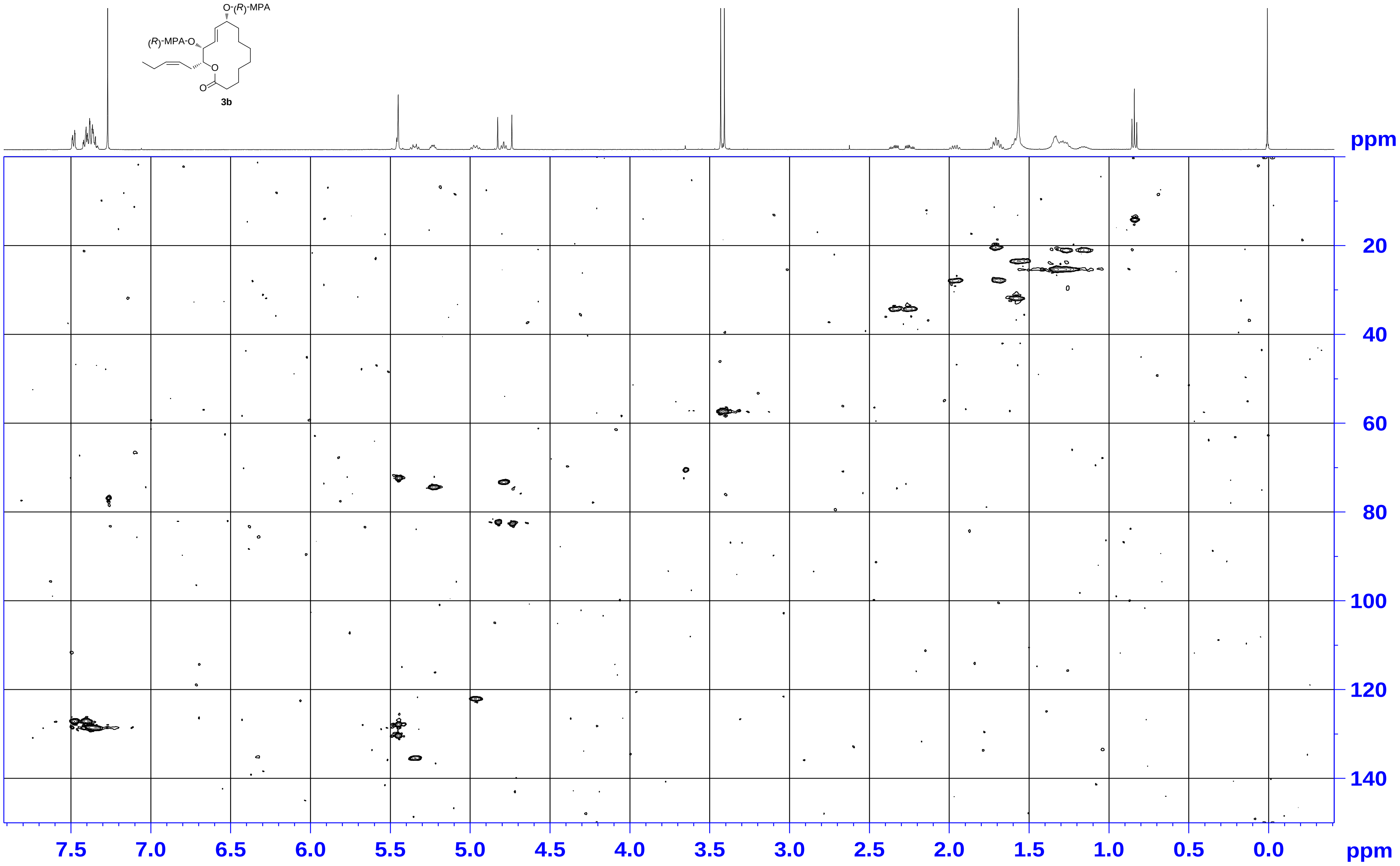

HSQC spectrum of *bis*-(*R*)-α-methoxyphenylacetic acid esters **3b** (500 MHz, $CDCl_3$).

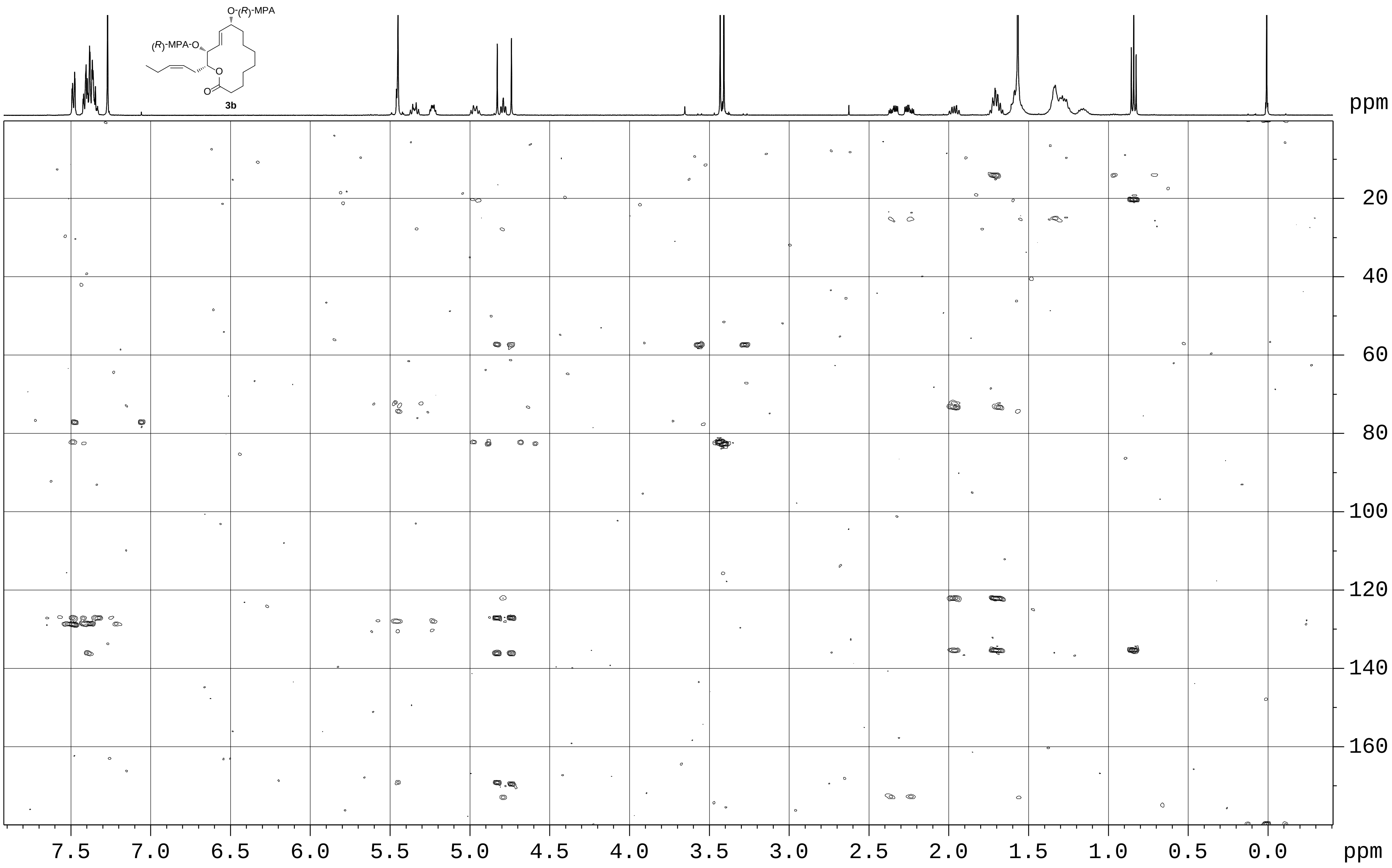

HMBC spectrum of *bis*-(*R*)-α-methoxyphenylacetic acid esters **3b** (500 MHz, $CDCl_3$).

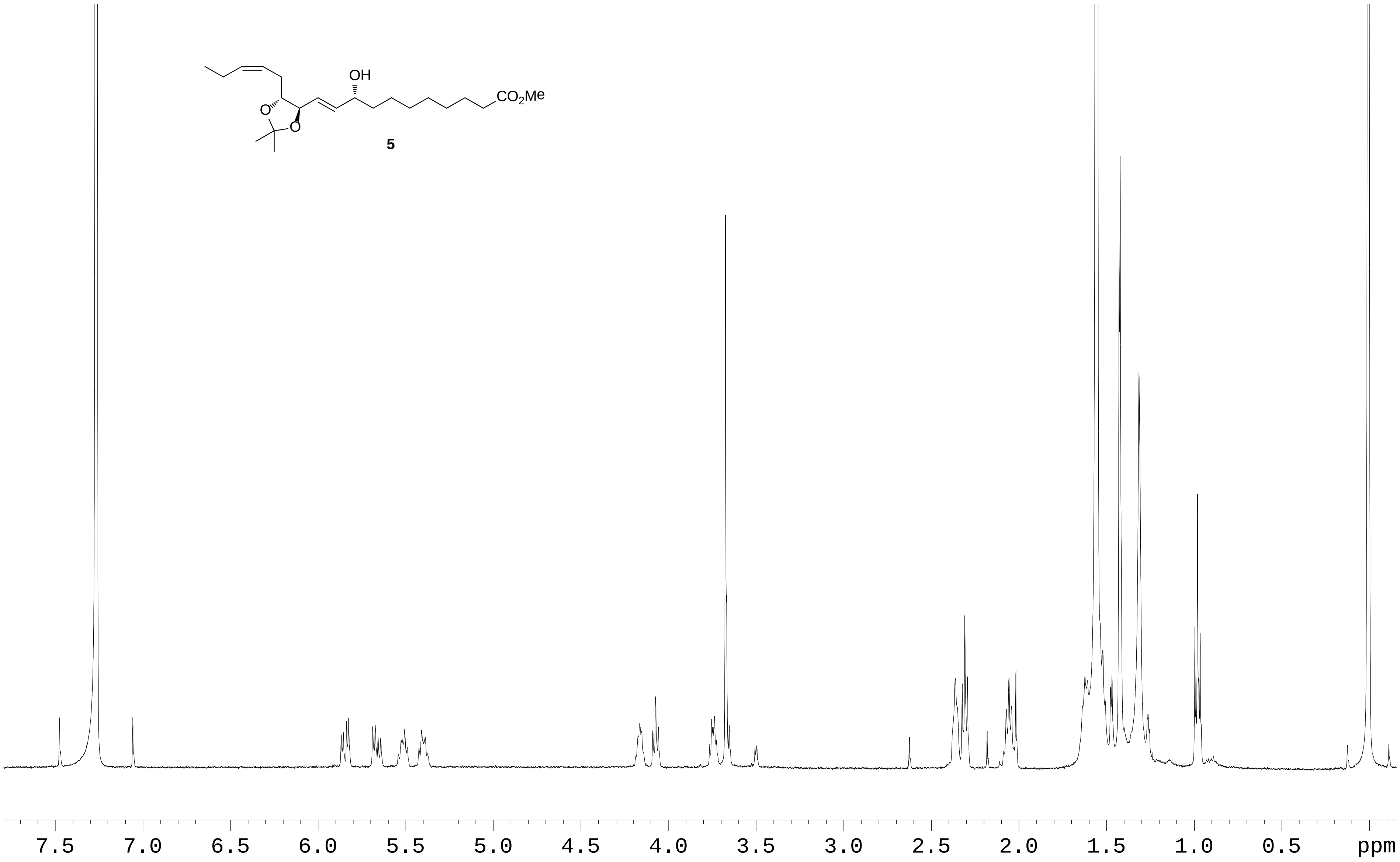

^{1}H-NMR spectrum of **5** (500 MHz, $CDCl_3$).

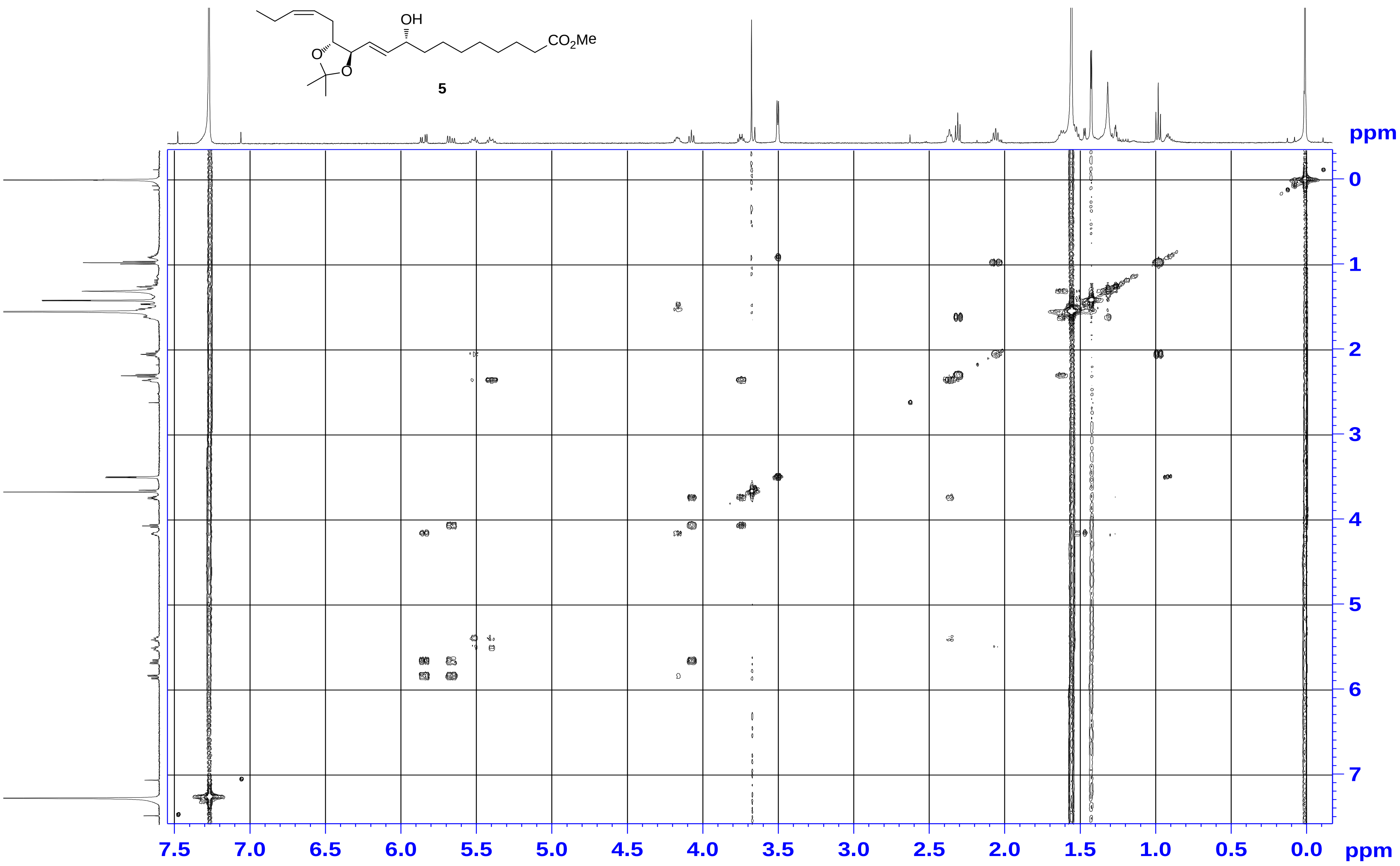

COSY spectrum of **5** (500 MHz, $CDCl_3$).

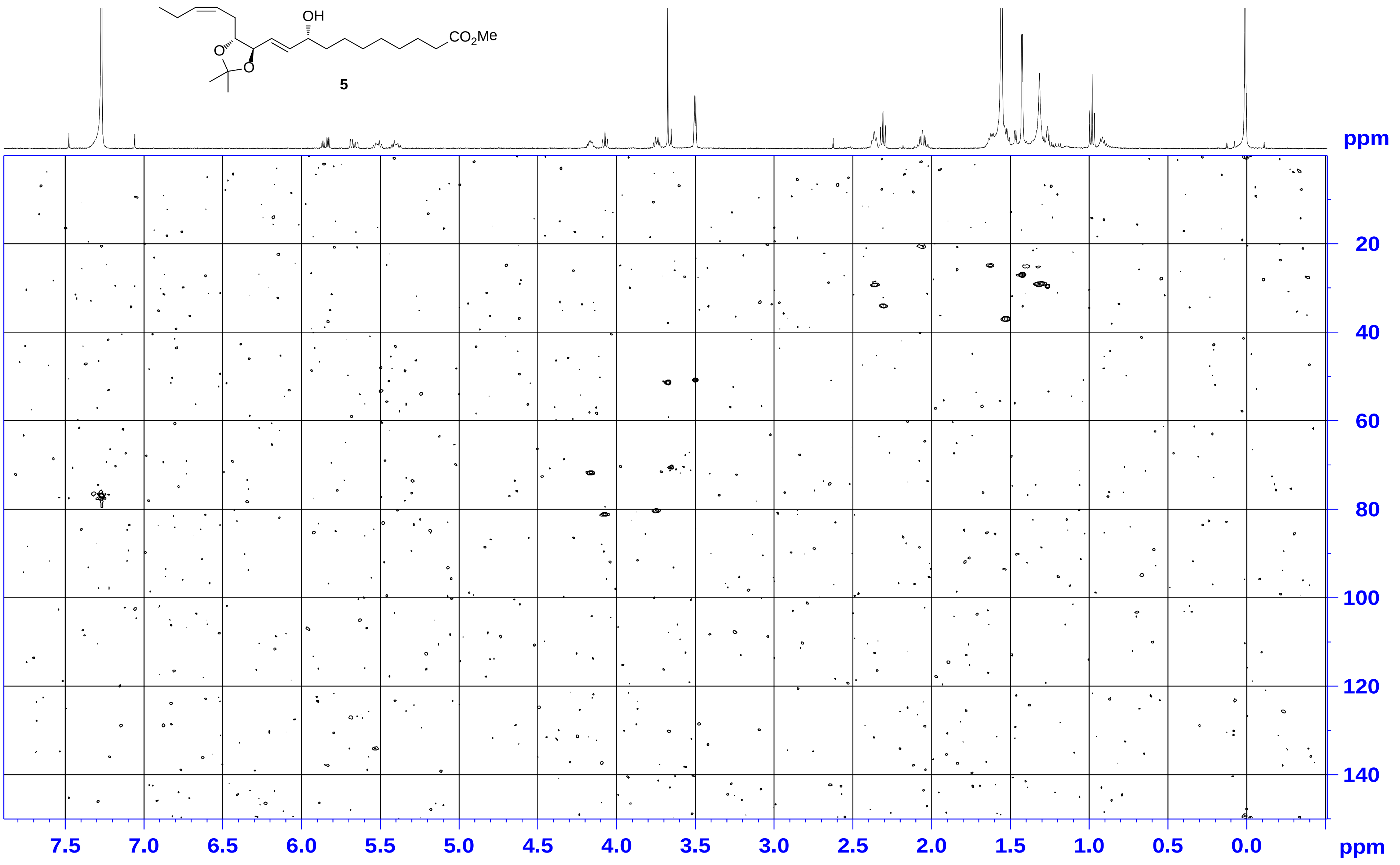

HSQC spectrum of **5** (500 MHz, $CDCl_3$).

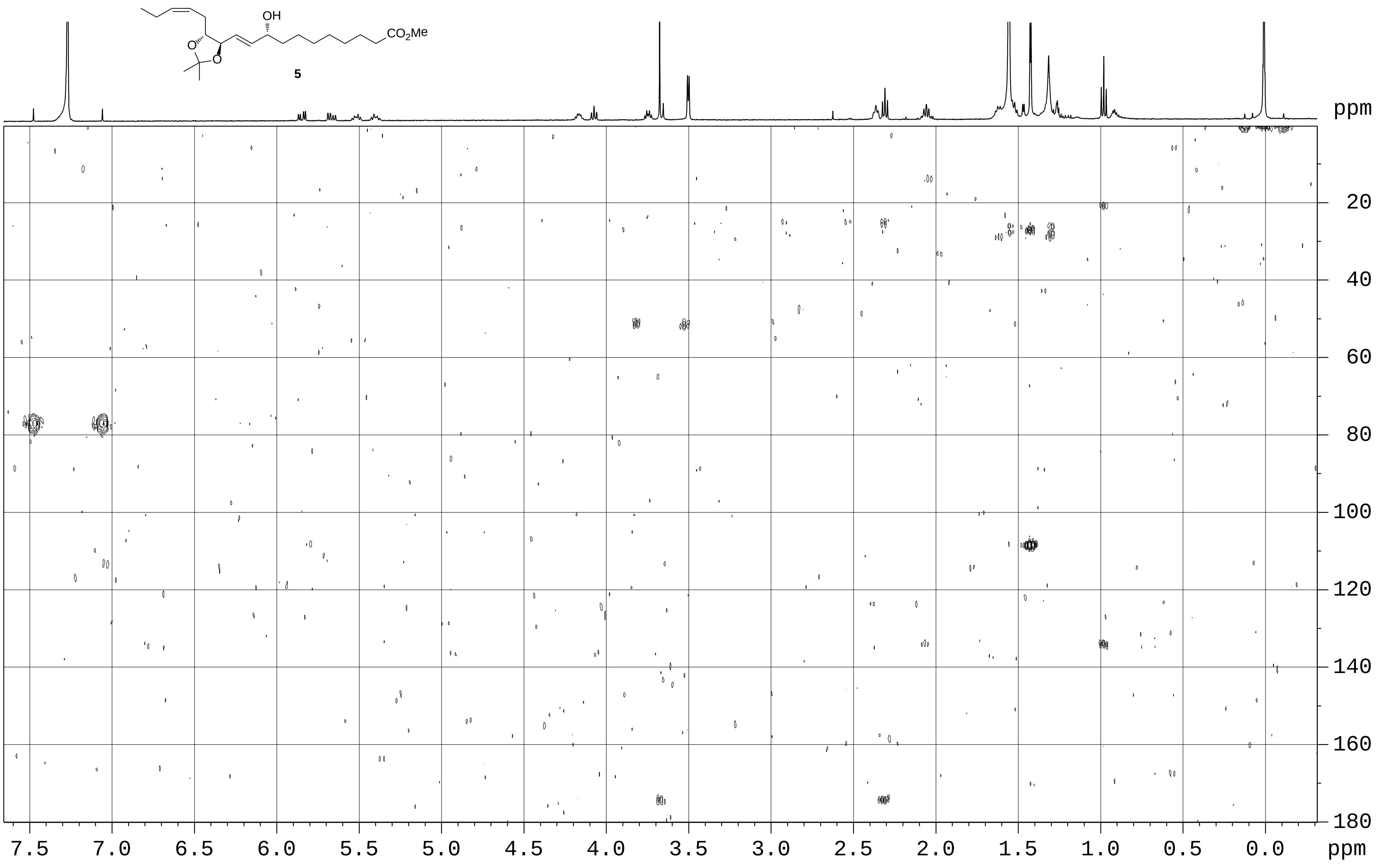

HMBC spectrum of **5** (500 MHz, $CDCl_3$).

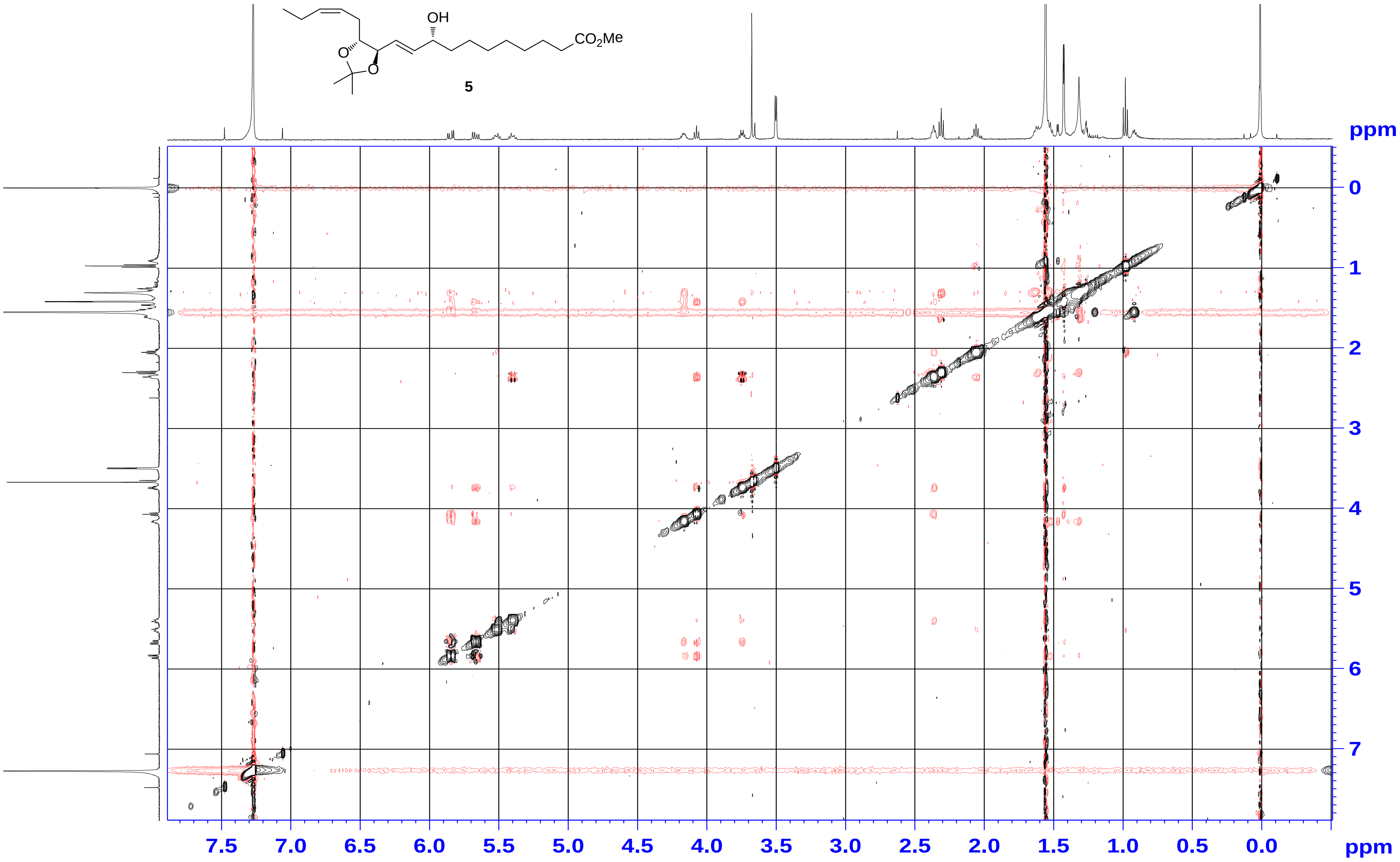

NOESY spectrum of **5** (500 MHz, $CDCl_3$).
